# Peptidylarginine deiminase 2 plays a key role in osteogenesis by enhancing RUNX2 stability through citrullination

**DOI:** 10.1038/s41419-023-06101-7

**Published:** 2023-08-30

**Authors:** Hyun-Jung Kim, Hye-Rim Shin, Heein Yoon, Min-Sang Park, Byung-Gyu Kim, Jae-I Moon, Woo-Jin Kim, Seung Gwa Park, Ki-Tae Kim, Ha-Neui Kim, Je-Yong Choi, Hyun-Mo Ryoo

**Affiliations:** 1 Department of Molecular Genetics and Dental Pharmacology, School of Dentistry and Dental Research Institute, Seoul National University, Seoul, South Korea; 2 Center for Genomic Integrity, Institute for Basic Science (IBS), Ulsan, South Korea; 3 Center for Musculoskeletal Disease Research and Center for Osteoporosis and Metabolic Bone Diseases, Department of Internal Medicine, Division of Endocrinology and Metabolism, University of Arkansas for Medical Sciences, Little Rock, Arkansas USA; 4 Department of Biochemistry and Cell Biology, Cell and Matrix Research Institute, BK21 Plus KNU Biomedical Convergence Program, Skeletal Disease Analysis Center, Korea Mouse Phenotyping Center, School of Medicine, Kyungpook National University, Daegu, South Korea

**Keywords:** Bone development, Post-translational modifications

## Abstract

Peptidylarginine deiminase (PADI) 2 catalyzes the post-translational conversion of peptidyl-arginine to peptidyl-citrulline in a process called citrullination. However, the precise functions of PADI2 in bone formation and homeostasis remain unknown. In this study, our objective was to elucidate the function and regulatory mechanisms of PADI2 in bone formation employing global and osteoblast-specific *Padi2* knockout mice. Our findings demonstrate that *Padi2* deficiency leads to the loss of bone mass and results in a cleidocranial dysplasia (CCD) phenotype with delayed calvarial ossification and clavicular hypoplasia, due to impaired osteoblast differentiation. Mechanistically, *Padi2* depletion significantly reduces RUNX2 levels, as PADI2-dependent stabilization of RUNX2 protected it from ubiquitin-proteasomal degradation. Furthermore, we discovered that PADI2 binds to RUNX2 and citrullinates it, and identified ten PADI2-induced citrullination sites on RUNX2 through high-resolution LC-MS/MS analysis. Among these ten citrullination sites, the R381 mutation in mouse RUNX2 isoform 1 considerably reduces RUNX2 levels, underscoring the critical role of citrullination at this residue in maintaining RUNX2 protein stability. In conclusion, these results indicate that PADI2 plays a distinct role in bone formation and osteoblast differentiation by safeguarding RUNX2 against proteasomal degradation. In addition, we demonstrate that the loss-of-function of PADI2 is associated with CCD, thereby providing a new target for the treatment of bone diseases.

## Introduction

Peptidylarginine deiminases (PADIs) are calcium-dependent hydrolases that convert peptidyl-arginine to peptidyl-citrulline, a process known as protein citrullination or deimination. This post-translational modification (PTM) can change the functions of the modified proteins, in terms of protein-protein interactions, protein stability, and subcellular localization, owing to the change from positive to neutral charges [1–4]. Citrullination regulates several important physiological processes such as early embryogenesis [5], pluripotency of stem cells [6], oligodendrocyte differentiation [7], osteoblast differentiation, and senescence [8]. Abnormal citrullination is closely associated with human diseases, including rheumatoid arthritis, multiple sclerosis, and cancer [9–11]. Five PADI isozymes (PADI1, 2, 3, 4, and 6) have been identified in mammals, which have different tissue distributions and overlapping substrates [12]. Our recent study showed that PADI2 is the most prevalent PADI isozyme in osteoblasts and mesenchymal stromal cells and that its downregulation following oxidative stress or *Padi2* knockdown inhibits osteoblast differentiation and induces cellular senescence [8]. However, the in vivo function of PADI2 in skeletal formation has not been reported.

Runt-related transcription factor 2 (RUNX2) is an essential master transcription factor in skeletogenesis [13]. *Runx2* knockout mice or C-terminal deletion mice exhibit a complete absence of mineralized bone in the calvaria and long bones, suggesting that RUNX2 is required for intramembranous and endochondral bone formation [13–15]. Haploinsufficiency of RUNX2 causes cleidocranial dysplasia (CCD) syndrome, characterized by incomplete closure of the fontanelle, hypoplasia of the clavicle, short stature, and supernumerary teeth in both humans and mice [15, 16]. The molecular mechanisms regulating RUNX2 expression and function have been extensively studied. In particular, RUNX2 PTMs, such as phosphorylation, acetylation, and ubiquitination, play important roles in their stability, DNA binding activity, and interactions with other proteins [17–20]. However, citrullination of RUNX2 by PADI enzymes and the effect of citrullination on its function have not yet been reported.

The aim of this study was to investigate whether PADI2 plays a role in bone formation and, if so, to determine the underlying mechanisms. Through comprehensive analyses of cells and tissues from mouse models with global *Padi2* deletion using *EIIA-Cre* mice or osteoblast-specific *Padi2* deletion using *Col1α1(2.3* *kb)-Cre* mice, we established critical roles of PADI2 in bone formation and homeostasis. Furthermore, we uncovered the underlying molecular mechanism by which PADI2 regulates osteoblast function, which may provide new insights into the pathogenesis of CCD and the development of therapeutics for bone abnormalities.

## Materials and methods

### Reagents, antibodies, and plasmids

Cycloheximide (C4859), Actinomycin D (A9515), and MG132 (M7449) were purchased from Sigma-Aldrich (St. Louis, MO, USA). Biotin-phenylglyoxal (a citrulline-specific probe-biotin) was obtained from Cayman Chemical (#17450; Ann Arbor, MI, USA). Antibodies against the following proteins were used in this study: PADI2 (66386-1-Ig; Proteintech, Rosemont, IL, USA), α-Tubulin (sc-8035; Santa Cruz Biotechnology, Inc., Dallas, Texas, USA), β-Actin (sc-47778; Santa Cruz Biotechnology), GAPDH (GTX100118; GeneTex), RUNX2 (D130-3; MBL Life science, Tokyo, Japan), DDK(FLAG) (Origene, Rockwille, MD, USA), HA (Cat# 901503; BioLegend, San Diego, CA, USA), and Strep-TagII (A01732S; GenScript, Piscateway, NJ, USA). Horseradish peroxidase-conjugated goat anti-mouse and goat anti-rabbit IgG secondary antibodies was purchased from GenDEPOT (Baker, TX, USA). The following siRNAs used in the present study were purchased from Origene: mouse Padi2 siRNA (SR418983B and C) and negative control siRNA (SR30004). Human PADI2 siRNA was purchased from BIONEER Corporation (11240-2; Daejeon, South Korea). An HA-ubiquitin (#18712) plasmid construct was obtained from Addgene (Cambridge, MA, USA). The Flag-PADI2-IRES-EGFP construct was a gift from Professor Karl-Henning Kalland at the University of Bergen, Norway [21]. For constructing Strep-Tag II-Runx2 plasmid, the coding region of mouse Runx2 transcript variant 1 (NM_001146038) was synthesized by Cosmogenetech Inc. (Daejeon, South Korea) and digested with NheI and BamHI and then cloned into Strep-Tag II vector. The full sequence was confirmed by Sanger sequencing. Recombinant human RUNX2 (RefSeq: NP_004339; Cat# TP760214) was purchased from Origene.

### Mice

To generate *Padi2* conditional knockout mice, *Padi2*^*fl/+*^ mice (C57BL/6J-Padi2^em1cyagen^) were purchased from Cyagen (Cat# CKOCMP-18600-Padi2-B6J-VA; Santa Clara, CA, USA). Exon 2 of *Padi2* was selected as the conditional knockout region (cKO region: ~684 bp), and CRISPR/Cas9-mediated deletion of this region resulted in a frameshift of the gene, leading to the loss of function of the mouse *Padi2* gene. *EIIA-Cre* (B6.FVB-TgN [EIIA-cre] C3739Lm) was obtained from Jackson Laboratory (Bar Harbor, ME). *Co11α1(2.3* *kb)-Cre* (B6D2F1) transgenic mice were kindly provided by Professor Je-Young Choi (Kyungpook National University, Daegu, Korea) [22, 23]. To generate global *Padi2* knockout mice, we crossed *Padi2*^*fl/fl*^ mice with *EIIA-Cre* mice, a transgenic line in which Cre-mediated recombination occurs in a wide range of tissues including germ cells that transmit genetic alterations to the progeny. *Padi2*^*fl/fl*^, *Padi2*^*fl/+*^, and *EIIA-Cre; Padi2*^*+/+*^ mice were used as littermate controls for this study. To generate osteoblast-specific *Padi2* deletion mice, *Padi2*^*fl/fl*^ mice were crossed with *Co11α1(2.3* *kb)-Cre* mice. *Padi2*^*fl/fl*^, *Padi2*^*fl/+*^, and *Co11α1(2.3* *kb)-Cre; Padi2*^*+/+*^ mice were used as littermate controls. All the mice were maintained under specific pathogen-free conditions in individual ventilation systems. All animal experiments were reviewed and approved by the Institutional Animal Care and Use Committee and Special Committee on Animal Welfare, Seoul National University, Seoul, South Korea.

### Micro-computed tomography (μ-CT) analysis

The mice were euthanized by CO_2_ inhalation, and the dissected limbs were fixed with 4% paraformaldehyde. To acquire micro-CT scans of the femurs, we used a SKYSCAN 1273 (Bruker, North Billerica, MA). Femoral bone analysis was performed using a CT Analyzer (CTAn) (Bruker) and three-dimensional (3D) visualization was performed using CTVox (Bruker).

### Histological analysis

Specimens were fixed in 4% paraformaldehyde for 24 h for hematoxylin and eosin (H&E) staining and immunohistochemistry (IHC) of the limbs. P7 mice required decalcification for 24 h with 10% EDTA (pH 7.4) solution, and 4 weeks- and 4 months-old mice required decalcification for at least 5 days. After decalcification, dehydration, and paraffin infiltration were performed using an automated tissue processor (TP1020, Leica). Embedded tissues were cut to 6-µm-thick sections with a rotary microtome (RM2145, Leica). For IHC, the section slides were subjected to antigen retrieval in citrate buffer for 10 min at 90 °C. Primary antibodies to the following antigens were used: PADI2 (Proteintech), RUNX2 (MBL Life science), and Collagen type I (COL1) (sc-59772; Santa Cruz Biotechnology, Inc). All the stained images were acquired using a DP72 digital microimaging camera (Olympus) under a BX51 microscope (Olympus).

### Skeletal whole mount staining

Newborn specimens were deskinned, eviscerated, and fixed in 99.9% ethanol (EtOH) for 5 days at room temperature (RT). After rinsing with deionized water, staining was performed in a solution of 17 volumes of 70% EtOH, 1 volume of 99.9% acetic acid, 1 volume of 0.3% Alcian blue 8GS (A3157; Sigma-Aldrich) in 70% EtOH and 1 volume of 0.1% Alizarin red S (A5533; Sigma-Aldrich) in 95% EtOH for 2 days at RT. The samples were rinsed in deionized water and cleaned in 1% potassium hydroxide (KOH) for 3 days, followed by 1 week in 0.8% KOH-20% glycerol. The samples were transferred to 50, 80, and 100% glycerol for long-term storage.

### Tartrate-resistant acid phosphatase (TRAP) staining

To measure TRAP activity, femoral tissue sections and osteoclasts differentiated from BMMs were stained using a TRAP staining kit (PMC-AK04F; COSMO BIO Co. Ltd., Tokyo, Japan) according to the manufacturer’s protocol. For counterstaining, the slides were stained with Alcian blue solution to quantify the number of TRAP-positive multinucleated cells. The samples were analyzed using ImageJ software (National Institutes of Health, USA).

### Primary cell isolation

Primary mouse osteoblasts were isolated from the cranial bones of newborn - P3 mice. The dissected frontal and parietal bones were sliced and incubated for 30 min with trypsin/EDTA (SH30042.01; HyClone Laboratories Inc., Logan, UT) and type II collagenase (LS004176; Worthington Biochemical Corp.), respectively. Fibroblasts adhering to the calvarial bone and debris were washed off, and the sliced bones were incubated with type II collagenase for an additional 1 h. The collected cells were filtered and counted for use in further experiments or stored. To isolate bone marrow macrophages (BMMs), bone marrow cells were first isolated by washing the bone marrow of the forelimb and hindlimb bones of 2-month-old mice using a 1 mL syringe. The obtained bone marrow cells were treated with RBC lysis buffer Hybri-MaxTM (R7757; Sigma-Aldrich) and plated overnight. After 24 h, the cells in the supernatant were collected and treated in cell culture dishes containing 20 ng/ml M-CSF (315-02; Pepro Tech Inc., Rocky Hill, NJ, USA) for 5 days, and only the attached cells were collected.

### Cell culture

MC3T3-E1 cells, primary calvarial osteoblasts, CRISPR-Cas9-medicated MC3T3-E1 *Padi2* KO cells (#3-4 and #5-6) [8] were cultured in α-MEM with 10% fetal bovine serum (FBS) containing 100 U/mL penicillin and 100 µg/mL streptomycin in a 5% CO_2_ humidified atmosphere at 37 °C. To induce osteoblast differentiation, we used α-MEM growth medium supplemented with 10 mM β-glycerophosphate and 50 µg/mL ascorbic acid. The medium was changed every 2–3 days. 293 T cells were cultured in high-glucose Dulbecco’s modified Eagle’s medium (DMEM) with 10% fetal bovine serum (FBS) containing 100 U/mL penicillin and 100 µg/mL streptomycin. Human mesenchymal stem cells (hMSC) were purchased from STEMCELL Technologies (Vancouver, Canada) and cultured in accordance with the manufacturer’s protocol. For osteoclast differentiation, BMMs were seeded (3 × 10^4^ cells/300 ul/ well in a 96-well plate) and differentiated into osteoclasts using 20 ng/ml M-CSF and 80 ng/ml recombinant murine sRANKL (Pepro Tech, Rocky Hill, NJ, USA) for 5 days. All cell lines used in the study were confirmed to be free of mycoplasma contamination.

### siRNA transfection and plasmid transfection

For siRNA experiments, cells were cultured to approximately 80–90% confluence and then transfected with 20 nM siRNA using Lipofectamine RNAiMax reagent (#13778; Invitrogen, Carlsbad, CA, USA) according to the manufacturer’s instructions. 293 T cells were transfected with the indicated plasmids using Lipofectamine^TM^ 2000 reagent (#11668019; Invitrogen) according to the manufacturer’s instructions. MC3T3-E1 cells were transfected with the indicated plasmids using the Neon^TM^ Transfection System (MPK5000; Invitrogen) according to the manufacturer’s instructions. hMSCs were transfected with the indicated plasmids using the TransIT^®^-LT1 Transfection Reagent (Mirus Bio LLC, WI, USA) according to the manufacturer’s instructions.

### Alkaline phosphatase (ALP) staining

The detailed procedure for each cell culture is explained above and is shown in the figure legends. ALP staining was performed using alkaline phosphatase staining kit (Cat# AK20; COSMO BIO Co. Ltd) according to the manufacturer’s instructions. Briefly, cells were carefully rinsed with 1xPBS and fixed with 4% paraformaldehyde at room temperature for 15 min. After fixation, the cells were rinsed with PBS and incubated in the BCIP/NBT liquid substrate for 30 min to 2 h. The color change was monitored, and the reaction was stopped by washing with PBS. Stained cell cultures were imaged using FUSION FX (VILBER, France). All staining data were obtained from three independent experiments.

### Alizarin red S (ARS) staining

The detailed cell culture procedure is explained in the previous section and figure legends. After the induction of osteoblast mineralization, the cells were fixed with 4% paraformaldehyde at room temperature for 15 min. Cells were rinsed with 1xPBS and stained with 500 μL 0.5% alizarin red stain solution, pH 4.2, for 30 min at room temperature. After incubation, the cells were rinsed with ddH_2_O on an orbital shaker for 5 min, three times. The mineralized nodules were stained as red spots after removing the unincorporated excess dye with ddH_2_O. Plates were scanned with FUSION FX (VILBER, France). All staining data were obtained from three independent experiments.

### Western blot analysis

Protein lysates were prepared using a PRO-PREP protein extraction solution (Cat#17081; iNtRON, South Korea) according to the manufacturer’s protocol. Equal amounts of protein were resolved by sodium dodecyl sulfate-polyacrylamide gel electrophoresis (SDS-PAGE) and transferred onto a polyvinylidene fluoride (PVDF) membrane. After blocking with 5% nonfat skim milk, the membrane was blotted with the designated primary and secondary antibodies, developed using the enhanced chemiluminescence method (Clarity™ Western ECL Substrate, #170-5060; Bio-Rad), and visualized using FUSION FX (VILBER, France). GAPDH, α-Tubulin, or β-Actin were used as a protein loading control. The full length uncropped original western blots were uploaded as a single supplemental material file.

### RNA preparation and quantitative real-time PCR

Total RNA was isolated using an RNeasy Mini Kit (Qiagen, Hilden, Germany). Total RNA (1 μg) was reverse-transcribed into cDNA using the PrimeScript RT Master Mix (Perfect Real Time) (RR036A; TaKaRa, Japan) and real-time quantitative PCR (qPCR) was performed using the TB Green® Premix Ex Taq^TM^ (RR420A; TaKaRa) on a StepOnePlus^TM^ Real-Time PCR System (Applied Biosystems^TM^). The following thermal conditions were used for real-time PCR: 95 °C for 1 min, followed by 40 cycles of 95 °C for 15 s and 60 °C for 30 s. The primers used in this study are listed in Supplementary Table 1. The relative expression levels were calculated with the 2^−*ΔΔCT*^ method [24]. The reactions were performed using three replicate samples from three independent experiments.

### Immunofluorescence staining and confocal microscopy

Cells grown on coverslips were fixed in 4% paraformaldehyde, blocked, and incubated with primary and corresponding secondary antibodies (Alexa Fluor 488- or 568-conjugated) (A11034; Invitrogen). A mounting medium containing DAPI was used to visualize the nuclei. The cells were examined under a confocal microscope (LSM 800, Carl Zeiss), and representative cells were selected and imaged.

### Co-immunoprecipitation

For immunoprecipitation, the cells were lysed in PRO-PREP protein extraction solution (iNtRON). Then cell extracts were incubated with MagStrep “type3” XT beads (Cat# 2-4090-002; IBA Lifesciences GmbH, Germany) overnight at 4 °C with constant rotation. Immunocomplexes were washed four times with IP lysis buffer (25 mM Tris-HCl (pH7.4), 150 mM NaCl, 1% NP-40, 1 mM EDTA, and 5% glycerol), and subsequently resolved by SDS-PAGE followed by western blot analysis.

### Ubiquitination assay

To detect ubiquitinated RUNX2, 293 T cells were co-transfected with the plasmids indicated in each experiment. The cells were then treated with 20 μM MG132 for 6 h on 3 days after transfection. Whole cell lysates prepared with PRO-PREP protein extraction solution were immunoprecipitated with Strep-Tag II magnetic beads and then resolved by SDS-PAGE followed by western blot analysis.

### Protein half-life assay

MC3T3-E1 cells were electroporated with the plasmids indicated in individual experiments, changed with osteogenic medium 48 h after electroporation, and additionally incubated for 3 days. On day 3, 20 ug/mL cycloheximide was treated and incubated for 0, 3, and 6 h.

### mRNA half-life assay

MC3T3-E1 cells were electroporated with the plasmids indicated in the individual experiments, changed with osteogenic medium 48 h after electroporation, and additionally incubated for 3 days. On day 3, 4 μg/mL Actinomycin D was treated and incubated for 0, 3, and 6 h.

### Site-directed mutagenesis

In-Fusion® HD Cloning kit (Cat# 639648; TAKARA) was used to generate site-directed mutagenesis of Runx2 citrullination sites according to the manufacturer’s instructions. The Strep-mRunx2 construct was used as a template for arginine (R) to lysine (K) mutagenesis at ten Runx2 citrullination sites: R25K, R26K, R188K, R193K, R197K, R232K, R235K, R236K, R381K, and R393K. The oligonucleotide sequences for each mutagenesis are listed in Supplementary Table 2. Full sequences of the mutant constructs were confirmed using Sanger sequencing.

### In vitro citrullination, biotin-phenylglyoxal (Biotin-PG) labelling, and detection of citrullinated RUNX2

For in vitro citrullination assay, 0.5 μg recombinant human RUNX2 protein (rhRUNX2) (Origene) and one unit recombinant human PAD2 (#10785; Cayman Chemical, Ann Arbor, MI, USA) were incubated with reaction buffer (50 mM HEPES (pH7.5), 5 mM DTT, 10 mM CaCl_2_, 50 mM NaCl) at 37 °C for 2 h. After in vitro citrullination, the samples were labeled with Biotin-PG (Cayman) according to previously reported methods [25, 26]. Briefly, 19.5 μL samples (0.5 μg) were incubated with 20% trichloroacetic acid (5 μL of 100% TCA) and 0.1 mM Biotin-PG (0.5 μL of 5 mM stock) for 30 min at 37 °C. After 30 min of incubation, the reaction was quenched with citrulline dissolved in 50 mM HEPES pH7.6 (final concentration, 100 mM). Proteins were precipitated by placing the reaction mixture on ice for 30 min followed by centrifugation (14,000 rpm, 15 min) at 4 °C. The supernatant was removed, and the protein pellet was washed twice with cold acetone and dried. Proteins were resuspended in neutral resuspension buffer (50 mM HEPES, pH 8.0, containing 100 mM arginine). 6x SDS loading dye was added and the samples were boiled for 10 min. The samples are sonicated for 2–5 s. The samples were separated by SDS-PAGE and transferred to PVDF membranes. The membranes were blocked with 5% BSA in PBS for 1 h at room temperature, incubated with streptavidin-HRP in blocking solution for 10 min at room temperature, washed with PBS for 5 min three times, and washed with dH_2_O for 5 min. Membranes were developed using an enhanced chemiluminescence method (Clarity™ Western ECL Substrate, Bio-Rad) and visualized using FUSION FX.

### Identification of RUNX2 citrullination sites by mass spectrometry analysis

rhRUNX2 (Origene) and citullinated rhRUNX2 were separated by SDS-PAGE and Coomassie staining with Imperial™ Protein Stain (Thermo Fisher Scientific), respectively. For LC-MS/MS analyses, the gel was de-stained and the bands were cut and processed as follows. Briefly, purified protein bands were divided into 10 mm sections and subjected to in-gel digestion with trypsin /Lys-C. Tryptic digests were separated by online reversed-phase chromatography using a Thermo Scientific Eazy nano LC 1200 UHPLC equipped with an auto-sampler using a reversed-phase peptide trap Acclaim PepMapTM 100 (75 μm inner diameter, 2 cm length) and a reversed-phase analytical column PepMapTM RSLC C18 (75 μm inner diameter, 15 cm length, 3 μm particle size), both from Thermo Scientific. This procedure was followed by electrospray ionization at a flow rate of 300 nl/ min. The samples were eluted using a split gradient of 3–50% solution B (80% ACN with 0.1% FA) for 60 min and 50–80% solution B for 10 min, followed by washing with 100% solution B for 10 min. The chromatography system was coupled with an Orbitrap Fusion Lumos mass spectrometer. The mass spectrometer was operated in data-dependent mode with a 120,000 resolution MS1 scan (375–1500 m/z), an AGC target of 5e5, and a maximum injection time of 50 ms. Peptides above threshold 5e3 and charges 2–7 were selected for fragmentation with dynamic exclusion after 1 time for 15 s and 10 ppm tolerance. Mass spectrometry proteomics data were deposited to the ProteomeXchange Consortium via the PRIDE partner repository with dataset identifiers PXD040179 and 10.6019/PXD040179.

### Database search

Tandem mass spectra were extracted using [unknown] version [unknown]. Charge-state deconvolution and deisotoping were not performed. All MS/MS samples were analyzed using Sequest (Thermo Fisher Scientific, San Jose, CA, USA; version IseNode in Proteome Discoverer 2.4.1.15) and X! Tandem software (GPM, version X! Tandem Alanine (2017.2.1.4)). Sequest was set up to search Citrullinated proteins. fasta (unknown version, 12 entries) assuming the digestion enzyme trypsin. X! Tandem was set up to search a reverse concatenated subset of the Citrullinated proteins database (unknown version, 24 entries) also assuming trypsin. Sequest and X! Tandem were searched with a fragment ion mass tolerance of 0.60 Da and a parent ion tolerance of 5.0 PPM. Carbamidomethyl of cysteine was specified in Sequest and X! Tandem as a fixed modification. Deamidated of asparagine and arginine, oxidation of methionine, acetyl of lysine, and phospho of serine were specified in Sequest as variable modifications. Glu->pyro-Glu of the n-terminus, ammonia-loss of the n-terminus, Gln->pyro-Glu of the n-terminus, deamidated of asparagine and arginine, oxidation of methionine, acetyl of lysine and phospho of serine were specified in X! Tandem as variable modifications.

### Criteria for protein identification

Scaffold (version Scaffold_4.11.0, Proteome Software Inc., Portland, OR) was used to validate MS/MS based peptide and protein identifications. Peptide identifications were accepted if they could be established at greater than 95.0% probability. Peptide Probabilities from X! Tandem were assigned by the Scaffold Local FDR algorithm. Peptide Probabilities from Sequest were assigned by the Peptide Prophet algorithm [27] with Scaffold delta-mass correction. Protein identifications were accepted if they could be established at greater than 95.0% probability and contained at least 2 identified peptides. Protein probabilities were assigned by the Protein Prophet algorithm [28]. Proteins that contained similar peptides and could not be differentiated based on MS/MS analysis alone were grouped to satisfy the principles of parsimony.

### Statistical analysis

For statistical analyses, *P* values were calculated by unpaired two-tailed Student’s *t-*test (when comparing only two groups), one-way ANOVA, or two-way ANOVA (when comparing more than two groups) using GraphPad Prism 9. All results are expressed as the mean ± SD, and differences were considered significant at *P* < 0.05. *P* values are as follows: *P < 0.05; **P < 0.01; ***P < 0.001; ****P < 0.0001. To ensure data reliability, all experiments were performed as at least two or three independent experiments with three replicates. Representative results are shown in the figures.

## Results

### *Padi2*-deficient mice displayed decreased bone mass

**Fig. 1:**
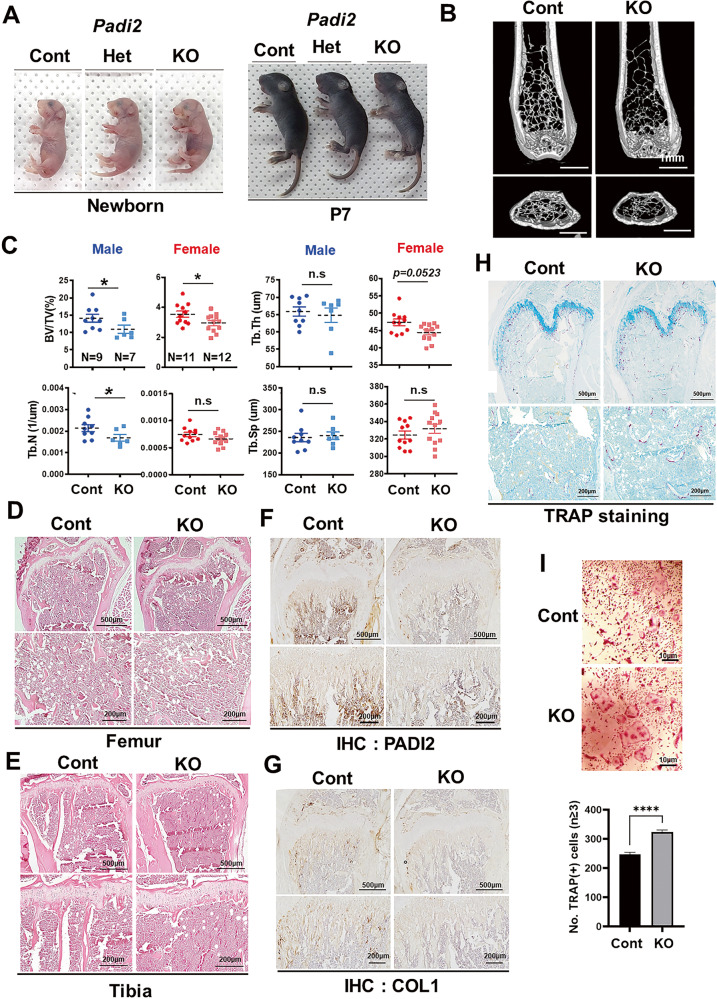
*Padi2* knockout mice exhibited reduced bone mass. **A** Physiognomy of *Padi2* control (Cont), hetero (Het), and knockout (KO) litters at newborn and P7 (*n* = 4–6 in each group). **B** Representative micro-CT images of the distal femur at 4 months in the arterial view of a coronal section and cross-sectional views are shown for each genotype (scale bar:1 mm). **C** Histomorphometric analyses of 3D micro-CT data of control and *Padi2* KO mice in both male (*n* = 9, Cont mice and *n* = 7, *Padi2* KO mice) and female (*n* = 11, Cont mice and *n* = 12, *Padi2* KO mice). BV/TV bone volume/tissue volume; Tb. Th trabecular thickness; Tb. N trabecular number; Tb. Sp trabecular separation. **D**, **E** Representative images of H&E stained distal femur and proximal tibiae from 4 months-aged control and *Padi2* KO mice (scale bar: 500 μm, 200μm). **F**, **G** Representative images of immunohistochemistry (IHC) for PADI2 (**F**) and type 1 collagen (COL1) at 4 months-aged Cont and KO mice (**G**) (scale bar: 500 μm, 200 μm). **H** Representative images of TRAP-stained trabecular bone of distal femur from 4 months-aged Cont and KO mice. TRAP-positive purple spots indicate multinucleated osteoclasts (scale bar: 500 μm, 200 μm); We performed two or three independent experiments with three biological replicates for each group (**D**–**H**). **I** Osteoclast identification by TRAP staining. Bone marrow-derived macrophages (BMMs) isolated from Cont and KO mice were differentiated into osteoclasts in the presence of CSF1 (20 ng/ml) and RANKL (100 ng/ml) for 5 days (scale bar: 10 μm). The graph shows the number of TRAP-positive osteoclasts with more than three nuclei (*n* = 8 in each group). Three independent experiments with eight biological replicates for each group. Data are expressed as the mean ± SE. *P < 0.05, **P < 0.01, ***P < 0.001, ****P < 0.0001. N.S, not significant.

Our recent study showed that PADI2 expression levels increased with osteoblast differentiation which was significantly inhibited by *Padi2* knockdown, indicating that PADI2 plays an important role in osteoblast differentiation [8]. To understand the role of PADI2 in the bone, we generated global *Padi2* knockout mice by crossing *Padi2*^*fl/fl*^ with *EIIA-Cre* mice, a transgenic line in which Cre-mediated recombination occurs in a wide range of tissues, including germ cells that transmit genetic alterations to the progeny [29]. Since *EIIA-Cre* mice had no significant effect on phenotypes including body size and bone structure compared to wild type mice based on accumulated in-house data and were not significantly different from *Padi2*^*fl/fl*^ and *Padi2*^*fl/+*^ mice in bone phenotype, all of *EIIA-Cre;Padi2*^*+/+*^, *Padi2*^*fl/fl*^ and *Padi2*^*fl/+*^ mice were used as littermate controls referred to as ‘control’. *EIIA-Cre*;*Padi2*^*fl/+*^ and *EIIA-Cre*;*Padi2*^*fl/fl*^ mice were named Het and KO mice, respectively. *Padi2* KO mice did not show severe phenotypic abnormalities compared to control mice at birth and survived normally. However, the physical size of *Padi2* KO mice tended to be smaller than that of their control littermates at the newborn stage and postnatal day seven (P7) (Fig. 1A), and this trend was observed up to 4–5 weeks of age, but there was no significant difference in size between the two groups as they aged further. Body weights of the control and KO groups were consistently similar after 6 weeks of age without significant differences (Supplementary Fig. 1A, B). In male mice, from 14 weeks of age, *Padi2* KO mice exhibited a slight but significant weight gain compared to the control (Supplementary Fig. 1A).

Whole-body Alizarin red/Alcian blue staining revealed no significant defects in the skeletal structures of newborn *Padi2* KO mice and respective controls, including the ribs, vertebrae, and limbs. However, delayed mineralization of the calvarial bones and hypoplasia of the clavicles were observed (Supplementary Fig. 2; Fig. 3A). Whole-body micro-computed tomography (μ-CT) analysis showed reduced cranial ossification and a significant decrease in femur length in P7-aged *Padi2* KO mice compared to their control littermates (Supplementary Fig. 3A, B). Tibial length exhibited a decreasing tendency in *Padi2* KO mice, although no statistically significant decrease was observed compared to the control group (Supplementary Fig. 3B, lower panel). Although PADI2 is the predominant isozyme among the five PADIs in mouse primary calvarial osteoblasts (pOBs) (Supplementary Fig. 4A), other isozymes may compensate for the effect of *Padi2* deficiency on skeletal bones. To determine whether other PADI isozymes compensate for the PADI2 deficiency, we compared the mRNA levels of PADI isozymes in pOBs isolated from *Padi2* control and KO mice. Interestingly, *Padi2* deficiency reduced the mRNA levels of other *Padi* isozymes (Supplementary Fig. 4B), indicating that the bone phenotypes seen in *Padi2* KO mice were not offset by the compensation from other PADI isozymes. To investigate the in vivo effects of PADI2 on the skeletal system of adult mice, we compared the changes in bone-related elements of the distal femur between 4-month-old *Padi2* control and KO mice using μ-CT. The *Padi2* KO mice showed significantly decreased trabecular bone mass compared to control in males and females (Fig. 1B, C). Trabecular bone per tissue volume (BV/TV) in *Padi2* KO mice was lower than that in control, which was accompanied by a reduction in trabecular number and thickness (Fig. 1C). A significant decrease in vertebral bone volume was also observed in 4-month-old *Padi2* KO mice, with fewer trabeculae and increased trabecular separation (Supplementary Fig. 5A, B). Long bone sections from 4-month-old *Padi2* control and KO mice were subjected to histological analyses. Hematoxylin and eosin (H&E) staining showed that trabecular bones in the femur and tibiae were largely reduced in *Padi2* KO mice compared to those in control (Fig. 1D, E). Immunohistochemistry (IHC) showed that while PADI2 was highly abundant in the osteoblasts of control mice, it was almost absent in the femur of *Padi2* KO mice (Fig. 1F). In addition, the level of type 1 collagen α1 (COL1A1), a representative osteoblast marker, was significantly lower in *Padi2* KO mice than in the control (Fig. 1G).

Tartrate acid resistant phosphatase (TRAP) staining was performed to assess the effects of *Padi2* knockout on osteoclast. TRAP-positive multinucleated cells were significantly increased on the surface of the trabecular bone in P7- and 4-month-old *Padi2* KO mice compared to that in the control (Supplementary Fig. 6A; Fig. 1H). To investigate whether the increased osteoclastogenesis in *Padi2* KO mice was due to *Padi2* deficiency, bone marrow cells were collected from the femoral bones of 2-month-old *Padi2* control and KO mice and differentiated into osteoclasts. RT-qPCR data demonstrated that early and late osteoclast differentiation markers, including *Pu1*, *Nfatc1, Ctsk*, *Trap*, and *Mmp9* significantly increased in *Padi2* KO osteoclasts compared to those in control (Supplementary Fig. 6B). TRAP staining revealed that osteoclastogenesis in *Padi2* KO bone marrow macrophages (BMMs) was significantly increased compared to that from control BMMs (Fig. 1I). Based on these findings, *Padi2* deficiency results in significantly reduced bone mass due to impaired bone formation and increased osteoclastogenesis.

### Osteoblastic *Padi2* deficiency caused the reduced bone mass with impaired osteoblast differentiation

**Fig. 2:**
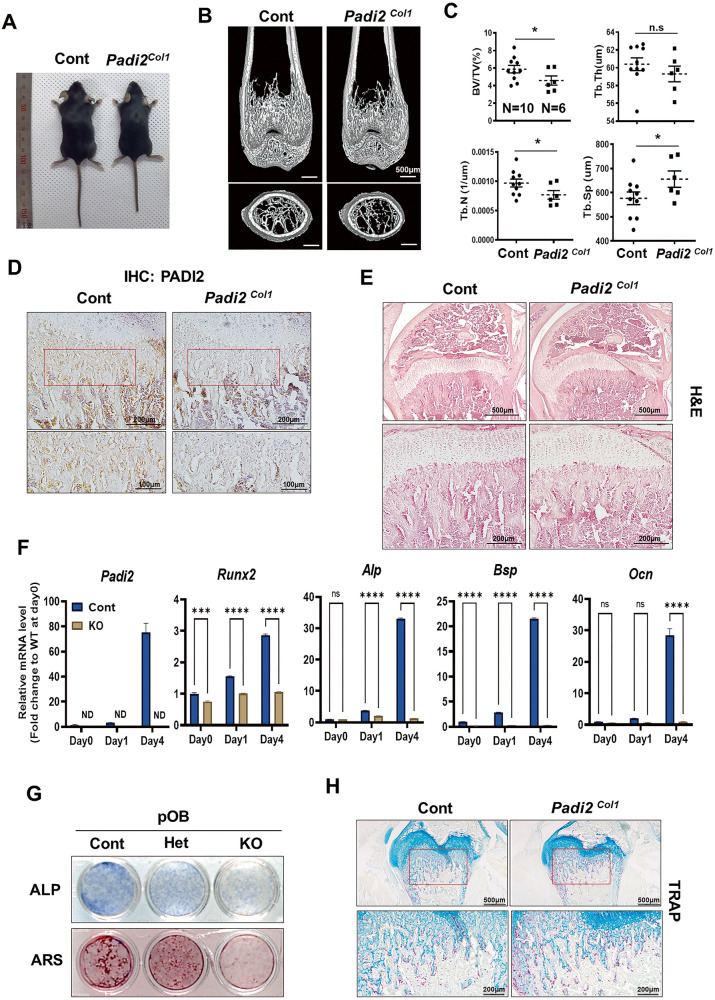
*Padi2* deficiency impaired osteoblast differentiation. **A** Representative view of 4-week-old female Cont and *Padi2-KO*^*Col1*^ mice. **B** Representative micro-CT images of the distal femur from 4-week-old female Cont and *Padi2*^*Col1*^ mice in midsagittal and coronal views. Scale bar: 500 μm. **C** Histomorphometric analysis of 3D micro-CT of the distal femur from data 4-week-old female Cont (*n* = 10) and *Padi2*^*Col1*^ (*n* = 6) mice. **D** IHC for PADI2 of distal femur from 4-week-old Cont and *Padi2*^*Col1*^ mice. Three independent experiments with three biological replicates for each group. Scale bar: 200 μm, 100 μm. **E** Representative images of H&E staining for distal femur from 4-week-old Cont and *Padi2*^*Col1*^ mice. Three independent experiments with three biological replicates for each group. Scale bar: 500 μm, 200 μm. **F** Relative mRNA expression of *Padi2* and bone marker genes in Cont and KO primary calvaria osteoblasts (pOBs) cultured or not (Day0) in osteogenic medium for each indicated day as determined by RT-qPCR. Three independent experiments with three biological replicates for each group. **G** ALP and ARS staining were performed in Cont, Het, and KO primary calvaria OBs cultivated in an osteogenic medium for 5 days and 21 days, respectively. Representative images from three independent experiments with two biological replicates for each group. **H** TRAP staining of distal femurs from 4-week-old Cont and *Padi2*^*Col1*^ mice. The boxed region is shown in higher magnification (bottom). Three independent experiments with three biological replicates for each group. Scale bar: 500 μm, 200 μm. Data are expressed as the mean ± SD. *P < 0.05, **P < 0.01, ***P < 0.001, ****P < 0.0001. N.S, not significant. ND not determined.

To further determine the role of PADI2 in bone formation, we generated osteoblast-specific *Padi2* knockout mice (hereafter referred to as *Padi2*^*Col1*^ mice) by crossing *Padi2*^*fl/fl*^ and *Col1α1(2.3* *kb)-Cre* mice, and *Col1α1(2.3* *kb)-Cre*;*Padi2*^*+/+*^, *Padi2*^*fl/fl*^ and *Padi2*^*fl/+*^ mice were all referred to as control. The body size of 4-week-old *Padi2*^*Col1*^ mice was smaller than that of the control (Fig. 2A). The μ-CT scan showed that 4-week-old *Padi2*^*Col1*^ mice displayed a significant decrease in bone mass compared to control (Fig. 2B). Further analysis indicated that BV/TV was significantly decreased in *Padi2*^*Col1*^ mice relative to the control, accompanied by a reduction in trabecular number and an increase in trabecular separation (Fig. 2C). Immunostaining of the distal femur confirmed that PADI2 was completely deficient in the osteoblasts of *Padi2*^*Col1*^ mice, while PADI2 expression was high in the osteoblasts of control mice (Fig. 2D). PADI2 was detected in the bone marrow cells of both the control and *Padi2*^*Col1*^ mice (Fig. 2D). These findings provide evidence of osteoblast-specific *Padi2* deficiency in *Padi2*^*Col1*^ mice. Consistent with the μ-CT data, H&E staining also showed diminished trabecular bone loss in the distal femur of *Padi2*^*Col1*^ mice compared to that in the control (Fig. 2E). Consistently, whereas *Padi2* and representative bone marker genes, including *Runx2*, *Alp*, *Bsp*, and *Ocn* showed an increase in pOB from the control group, in a differentiation stage-dependent manner, their mRNA levels were significantly decreased in the pOBs from *Padi2* KO mice (Fig. 2F). In addition, the osteoblast differentiation capability of pOBs isolated from the *Padi2* control, Het, and KO mice, correlated well with the *Padi2* gene dosage determined by alkaline phosphatase (ALP) and alizarin res S (ARS) staining (Fig. 2G). The lower the *Padi2* expression level, the greater the inhibition of osteoblast differentiation. Interestingly, *Padi2*^*Col1*^ mice also showed significantly increased numbers of TRAP-positive osteoclasts relative to control mice (Fig. 2H), indicating that secretory factors from *Padi2*-deficient osteoblasts may stimulate osteoclastogenesis. To determine whether *Padi2* deficiency induces osteoclastogenic factors in pOB, we examined the mRNA levels of *Csf-1*, *Rankl*, and *Opg* using RT-qPCR. *Csf-1* mRNA levels were significantly increased in pOBs from *Padi2* KO mice compared to those from the control (Supplementary Fig. 7A). An increased Rankl/Opg ratio was observed in the pOB from *Padi2* KO relative to that from the control owing to increased *Rankl* mRNA levels (Supplementary Fig. 7A). Our previous study showed that *Padi2* knockdown in MC3T3-E1 osteoblasts induced increased mRNA expression and secretion of CCL2, CCL5, and CCL7, which are known to promote osteoclastogenesis [8]. Similarly, the mRNA levels of *Ccl2*, *Ccl5*, and *Ccl7* were significantly increased in pOBs from *Padi2* KO compared to those from control (Supplementary Fig. 7B). Taken together, these results demonstrate that PADI2 is required for bone formation and osteoblast differentiation and that *Padi2* deficiency in osteoblasts can promote osteoclastogenesis.

### Ablation of *Padi2* causes a CCD-like phenotype

**Fig. 3:**
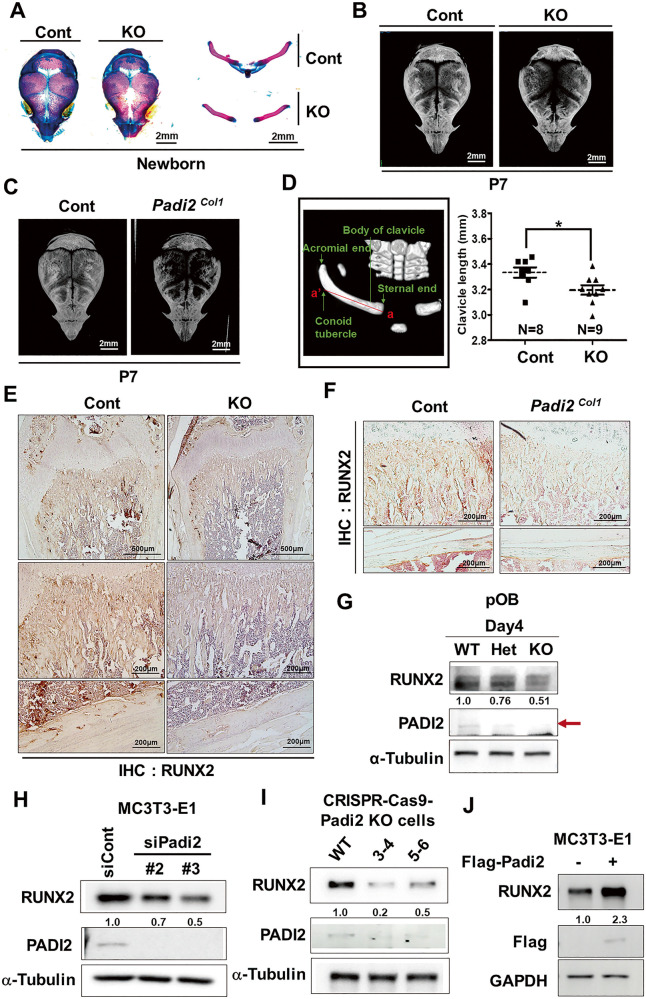
*Padi2* KO mice had CCD phenotype and showed the reduction of RUNX2 level. **A** Whole-mount skeleton staining of Cont (*n* = 6) and *Padi2* KO (*n* = 5) newborn littermates by Alizarin red and Alcian blue staining. Samples were cut into calvaria and clavicles. Scale bar: 2 mm. **B** Representative micro-CT images of the skull of postnatal day 7 (P7) Cont (*n* = 9) and *Padi2* KO (*n* = 9) mice. Scale bar: 2 mm. **C** Representative micro-CT images of the skull of P7-old Cont (*n* = 6) and *Padi2* ^*Col1*^ (*n* = 5) mice. Scale bar: 2 mm. **D** The landmarks of the mouse clavicle for measuring the length of the clavicle (left). The clavicle length (a-a’) was measured and graphed in each sample in P7 Cont (*n* = 8) and *Padi2* KO mice (*n* = 9) (right). **E** IHC for RUNX2 of the distal femur from 4-week-old Cont and *Padi2* KO mice. The second and third rows show enlarged areas of metaphysis and cortical bone, respectively. Three independent experiments with three biological replicates for each group. Scale bar: 500 μm, 200 μm. **F** IHC for RUNX2 of distal femur from 4-week-old WT and *Padi2*^*Col1*^ mice. The bottom shows cortical bone. Three independent experiments with three biological replicates for each group. Scale bar: 200 μm. **G** Primary calvarial OBs were cultured in an osteogenic medium for the indicated day, and RUNX2 and PADI2 levels were examined by western blot analysis. α-Tubulin was used as a loading control. RUNX2 level was quantified using ImageJ software and normalized with α-Tubulin. **H** MC3T3-E1 cells were transfected with scrambled control siRNA (siCont), *Padi2* siRNA #2 (siPadi2 #2), or *Padi2* siRNA #3 (siPadi2 #3) and then cultivated in osteogenic media for additional 3 days. RUNX2 and PADI2 levels were examined by western blot analysis. α-Tubulin was used as a loading control. RUNX2 level was quantified using ImageJ software and normalized with α-Tubulin. **I** RUNX2 and PADI2 levels in CRISPR–Cas9-mediated *Padi2* KO cell clones (#3–4 and #5–6) and control cells were examined by western blot analysis. α-Tubulin was used as a loading control. RUNX2 level was quantified using ImageJ software and normalized with α-Tubulin. **J** MC3T3-E1 cells were transfected with empty vector or *Flag-PADI2* plasmids and then cultivated in osteogenic media for an additional 3 days. RUNX2 and Flag-PADI2 levels were examined by western blot analysis. GAPDH was used as a loading control. RUNX2 level was quantified using ImageJ software and normalized with GAPDH. Western blot data was collected from at least two or three independent experiments; the representative results are shown here.

CCD is an autosomal-dominant human bone disease characterized by hypoplastic clavicles, patent fontanelles and sutures, and multiple other skeletal disorders [16]. As mentioned earlier, Alizarin red and Alcian blue staining showed that although newborn *Padi2* KO mice did not show significant defects in skeletal structures compared to the control, *Padi2* KO mice exhibited a CCD-like phenotype with hypomineralization of the calvarium and clavicular hypoplasia (Fig. 3A). In addition, hypomineralization of calvarial bones was confirmed in both global *Padi2* KO mice and osteoblast-specific *Padi2* deleted *Padi2*^*Col1*^ mice at P7 (Fig. 3B, C). The μ-CT scanning also confirmed that the clavicle lengths of *Padi2* KO mice, which were measured between the sternal end (a) and the conoid tubercle (a’) of the body of the clavicle, were significantly shorter than those of the control (Fig. 3D). CCD is genetically linked to a mutation in RUNX2 [30] and Runx2 haploinsufficiency is known to cause CCD. IHC demonstrated that compared to the control group, global *Padi2* KO mice and *Padi2*^*Col1*^ mice showed dramatically reduced RUNX2 levels in the trabecular and cortical bones of the distal femur (Fig. 3E, F). Primary osteoblasts from *Padi2* control, Het, and KO mice cultured in osteogenic media exhibited a substantial decrease in RUNX2 protein levels depending on the amount of PADI2 (Fig. 3G). Additionally, *Padi2* knockdown using *Padi2*-specific siRNA induced the significantly reduced RUNX2 levels (Fig. 3H). This phenomenon was also confirmed in the CRISPR–Cas9-mediated *Padi2* KO cell clones (#3-4 and #5-6) and WT control cells (Fig. 3I). In contrast, forced expression of *Padi2* significantly increased RUNX2 levels in MC3T3-E1 cells cultured in osteogenic medium (Fig. 3J). Collectively, these results suggest that *Padi2* deficiency in osteoblasts reduces RUNX2 protein levels, resulting in a CCD-like phenotype.

### PADI2 stabilized RUNX2 from ubiquitin-proteasomal degradation

**Fig. 4:**
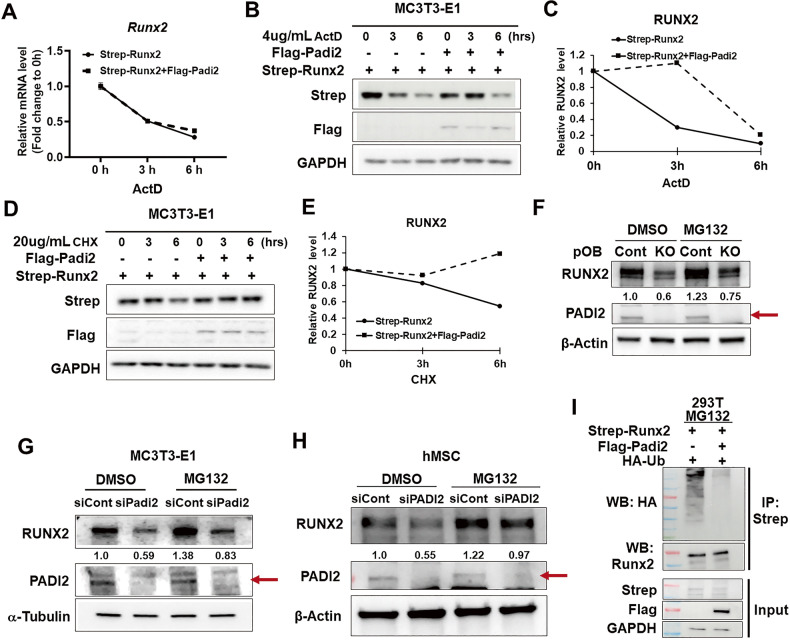
PADI2 protects RUNX2 from ubiquitin-proteasomal degradation pathway. **A**–**C** MC3T3-E1 cells were transfected with *Strep-Runx2* with or without *Flag-PADI2*, and then cultivated in osteogenic medium for 3 days. On day 3, 4 μg/mL Actinomycin D was treated and incubated for 0, 3, and 6 h. The half-life of *Runx2* mRNA and RUNX2 protein was determined by RT-qPCR (**A**) and western blot analysis (**B**), respectively. The intensities of Strep-RUNX2 protein levels were normalized against each GAPDH by ImageJ. The normalized values at 0 h were set as 1, and relative levels are shown (**C**). **D**, **E** MC3T3-E1 cells were transfected with *Strep-Runx2* together with or without *Flag-PADI2*, and then cultivated in osteogenic medium for 3 days. 20 μg/mL cycloheximide was treated on the last day and incubated for 0, 3, and 6 h. The half-life of RUNX2 protein was determined by western blot analysis (**D**). The intensities of Strep-RUNX2 protein levels were normalized against each GAPDH by ImageJ and relative levels are shown (**E**). **F** *Padi2* control and KO pOB cells were cultured in osteogenic media for 2 days, and 20 μM MG132 or DMSO as vehicle was treated for 6 h before harvesting cells, and western blot analysis followed. β-Actin was used as a loading control. RUNX2 level was quantified using ImageJ software and normalized with β-Actin. The red arrow indicates PADI2. **G** MC3T3-E1 cells were transfected with si*Cont* or si*Padi2* #2 and then cultivated in osteogenic media for an additional 2 days. 20 μM MG132 or DMSO as the vehicle was treated for 6 h before harvesting cells followed by western blot analysis. α-Tubulin was used as a loading control. RUNX2 level was quantified using ImageJ software and normalized with α-Tubulin. The red arrow indicates PADI2. **H** hMSCs were transfected with si*Cont* or si*PADI2* and then cultivated in osteogenic media for an additional 2 days. 20 μM MG132 or DMSO as the vehicle was treated for 6 h before harvesting cells followed by western blot analysis. β-Actin was used as a loading control. RUNX2 level was quantified using ImageJ software and normalized with β-Actin. The red arrow indicates PADI2. Western blot data were collected from at least two or three independent experiments; the representative results are shown here. **I** *Strep-Runx2*, *Flag-PADI2*, and *HA-ubiquitin* were transfected into 293 T cells. 3 days after transfection, cells were treated with 20 μM MG132 for 6 h, lysed, immunoprecipitated with Strep-Tag II magnetic beads, and immunoblotted with anti-HA or anti-RUNX2 antibody. Ubiquination assay was performed in three independent experiments; the representative results are shown here.

PADI2 is a post-translational modifying enzyme that converts peptidyl-arginine residues to citrulline via deimination, resulting in profound changes in the structure and function of target proteins including protein stability [2]. *Padi2* deficiency significantly decreased both RUNX2 mRNA and protein levels (Fig. 2F; Fig. 3E–I). To investigate whether PADI2 affected *Runx2* mRNA levels at the post-transcriptional level, MC3T3-E1 cells transfected with *Strep-Runx2* with or without *Flag-PADI2* were treated with Actinomycin D (ActD), a transcription inhibitor, during the indicated time points. RT-qPCR showed a similar decrease in *Runx2* mRNA levels after ActD treatment over time in both the control and PADI2 overexpression groups (Fig. 4A). However, the RUNX2 protein levels significantly decreased 3 h after ActD treatment in the group transfected with the empty vector, whereas it remained the same until 3 h after ActD treatment in the PADI2 overexpressing group (Fig. 4B, C). These results indicated that PADI2 is involved in the regulation of RUNX2 protein levels rather than *Runx2* mRNA stability. Next, we investigated whether PADI2 was involved in regulating the stability of RUNX2 by treatment with cycloheximide (CHX), a protein synthesis inhibitor. The RUNX2 protein level was maintained for up to 6 h after CHX treatment when *Padi2* was overexpressed. However, the RUNX2 level in the control group showed a time-dependent decrease after CHX treatment (Fig. 4D, E), indicating that PADI2 enhances the half-life of RUNX2 at the post-translational level. Next, we examined whether the *Padi2* depletion-induced decrease in RUNX2 was mediated by the ubiquitin-proteasome pathway. Treatment with MG132, a proteasome inhibitor, restored the reduced level of RUNX2 in *Padi2* KO pOB cells (Fig. 4F) as well as in cells with *Padi2* knockdown or CRISPR-Cas9-mediated *Padi2* knockout (Fig. 4G; Supplementary Fig. 8). This restorative effect was also observed in human mesenchymal stromal cells (hMSCs) (Fig. 4H), indicating that the role of PADI2 in regulating the stability of RUNX2 is consistent in human mesenchymal-derived cells. An ubiquitin-based immuno-precipitation assay confirmed that PADI2 overexpression drastically blocked the ubiquitination level of RUNX2 (Fig. 4I). Taken together, these results demonstrate that PADI2 contributes to the maintenance of RUNX2 stability by inhibiting ubiquitination-mediated proteasomal degradation of RUNX2.

### PADI2 citrullinates RUNX2

**Fig. 5:**
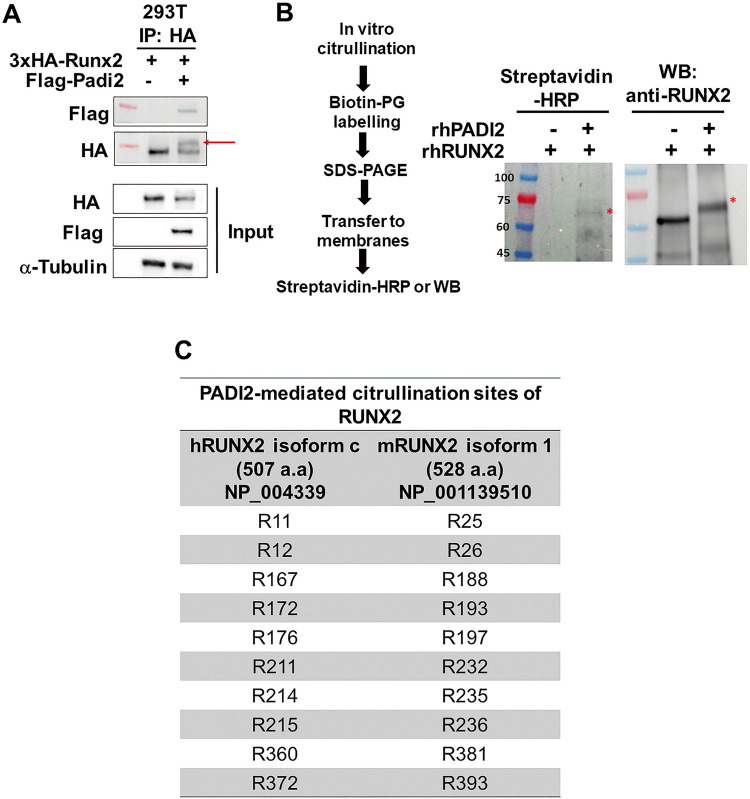
PADI2 citrullinates RUNX2 via physical interaction. **A** 293 T cells were transfected with 3x*HA-Runx2* together with or without *Flag-PADI2* and cultured for 3 days after the transfection. Cells were lysed, immunoprecipitated with anti-HA antibody and protein G-conjugated magnetic beads, and immunoblotted with indicated antibodies. The red arrow indicates predicted citrullinated RUNX2. Co-IP experiment was performed in three independent experiments; the representative results are shown here. **B** Workflow showing the detection of citrullinated RUNX2 by Biotin-PG labeling (left). Recombinant human RUNX2 (rhRUNX2) isoform c (NP_004339) was in vitro citrullinated by rhPADI2. The samples were labeled with Biotin-PG and then separated by SDS-PAGE followed by transfer to PVDF membrane. The membrane was incubated with Streptavidin conjugated with horseradish peroxidase (Streptavidin-HRP) or was immunoblotted with anti-RUNX2 antibody (right). A red asterisk indicates citrullinated RUNX2. This experiment was performed in three independent experiments; the representative results are shown here. **C** In vitro citrullinated rhRUNX2 isoform c was analyzed by LC-MS/MS. The ten arginine (R) sites of rhRUNX2 citrullinated by PADI2 were identified (left column). The mouse RUNX2 isoform 1 was used for site-directed mutagenesis of the 10 R sites and, for this, the R site in mouse RUNX2 isoform 1 matching the corresponding each R site in hRUNX2 isoform c is shown in the right column.

Since PADI2 protected RUNX2 from proteasomal degradation, we further investigated whether PADI2 interacted with RUNX2 for the citrullination of the protein. We transfected 293 T cells with *HA-Runx2* with or without *Flag-PADI2* plasmids and performed a co-immunoprecipitation (Co-IP) assay, which revealed a protein-protein interaction between PADI2 and RUNX2 (Fig. 5A). Interestingly, in the Co-IP experiment, PADI2 overexpression retarded the movement of some HA-tagged RUNX2 proteins (red arrow in Fig. 5A), suggesting citrullination of RUNX2 by PADI2. Next, to investigate whether PADI2 citrullinates RUNX2, recombinant human RUNX2 protein (rhRUNX2, NP_004339) was in vitro citrullinated by recombinant human PADI2 (rhPADI2) and then labeled with biotin-phenylglyoxal (Biotin-PG), a chemical probe that selectively binds to peptidyl-citrulline under acidic conditions [26, 31, 32]. Sequentially, the labeled proteins were subjected to SDS-PAGE and electro-transferred to the membranes. Streptavidin-HRP was used to detect citrullinated RUNX2 and western blot analysis was performed with an anti-RUNX2 antibody to confirm the presence of both citrullinated and non-citrullinated forms of RUNX2 (Fig. 5B, left panel). The in vitro citrullination assay showed that rhRUNX2 was citrullinated by rhPADI2, and the size of the citrullinated RUNX2 (cit-RUNX2, red asterisk) was slightly larger than that of non-citrullinated RUNX2 (Fig. 5B, middle and right panels). We investigated the effect of citrullination of RUNX2 by PADI2 on the function and fate of RUNX2. Initially, we determined the specific site of citrullination of RUNX2 by PADI2. To accomplish this, we generated cit-rhRUNX2 (NP_004339, 507 aa) by performing in vitro citrullination using rhPADI2. Subsequently we conducted in-gel digestion and high-resolution liquid chromatography-tandem mass spectrometry (LC-MS/MS) analyses to identify citrullination site (Supplementary Fig. 9A). Citrullinated forms of seven peptides containing R11, R12, R211, R214, R215, R360 and R372 were identified in the lower cit-RUNX2 band (blue arrow in Supplementary Fig. 9A), and three additional sites (R167, R172, and R176) were identified in the upper cit-RUNX2 band along with the above-mentioned seven sites (red arrow in Supplementary Fig. 9A), compared to native RUNX2 (Fig. 5C; Supplementary Fig. 9A, B). Manual interrogation of the high-resolution MS1 spectra confirmed the presence of the about 1 Da heavier citrullinated species for each of these peptides (Supplementary Fig. 9B). To assess the impact of these citrullination sites on the function and fate of RUNX2, we introduced substitutions in which each of the 10 arginine (R) sites citrullinated by PADI2 was replaced with lysine (K). This substitution was chosen because the conversion from R to K maintains a positive charge but prevents citrullination. Since mouse RUNX2 isoform 1 (mRUNX2, NP_001139510, 528 aa) is the osteoblast-specific isoform and its amino acid sequence is highly conserved with human RUNX2 isoform c, which has a high homology of approximately 95% (Supplementary Fig. 10), it was utilized for mutagenesis. In Fig. 5C, each R site in mouse RUNX2 isoform 1 that corresponds to the 10 citrullinated R sites in human RUNX2 isoform c is depicted, and these R sites were converted to K.

### PADI2-mediated citrullination of RUNX2 is required for the maintenance of RUNX2 stability

**Fig. 6:**
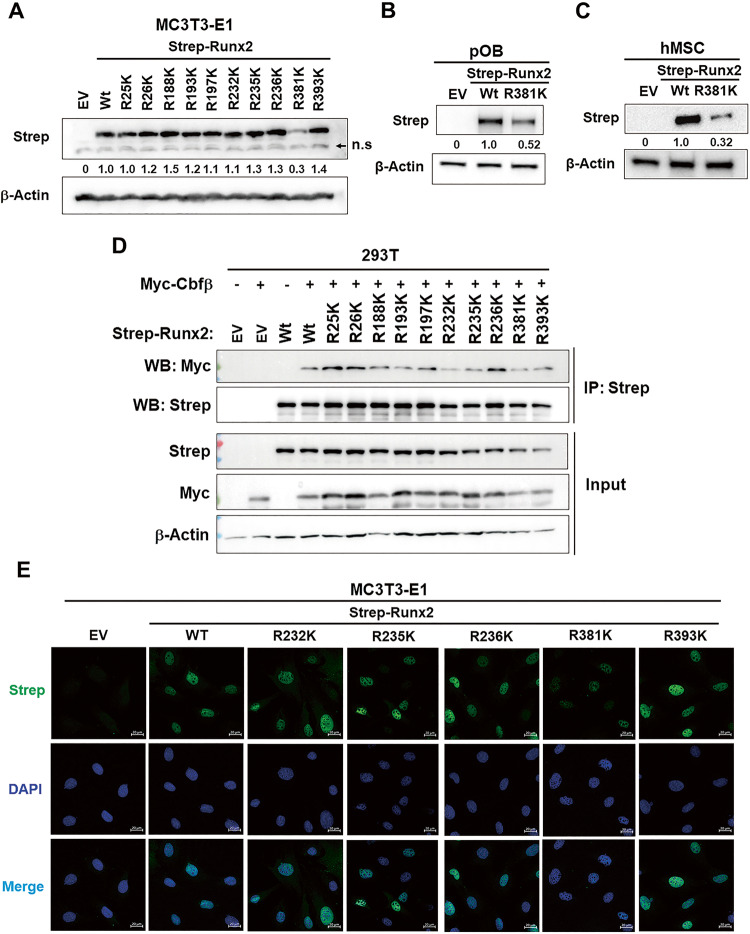
PADI2-catalyzed citrullination of RUNX2 is required for RUNX2 stabilization. **A** MC3T3-E1 cells were transfected with empty vector (EV), *Strep-Runx2* wild type (Wt), or R-to-K mutants and cultured in osteogenic media for 3 days after transfection. Cells were lysed and western blot analysis was performed. β-Actin was used as a loading control. Strep-RUNX2 level was quantified using ImageJ software and normalized with β-Actin. The arrow indicates non-specific bands (n.s). Western blot data were collected from three independent experiments; the representative results are shown here. **B**, **C** pOB cells and hMSCs were transfected with EV, *Strep-Runx2* WT, or R381K mutant plasmids and cultured in osteogenic media for 2 days. Cells were lysed and western blot analysis was performed. β-Actin was used as a loading control. Strep-RUNX2 level was quantified and normalized with β-Actin using ImageJ software. Western blot data were collected from two independent experiments; the representative results are shown here. **D** 293 T cells were transfected with *Strep-Runx2* Wt or R-to-K mutants with or without *Myc-Cbfβ* plasmids and then incubated for 3 days after transfection. Cells were lysed, immunoprecipitated with Strep-Tag II magnetic beads, and immunoblotted with indicated antibodies. Co-IP experiment was performed in three independent experiments; the representative results are shown here. **E** MC3T3-E1 cells were transfected with *Strep-Runx2* Wt or R-to-K mutants and cultured for 2 days after transfection. Cells were fixed with 4% PFA, permeabilized, and then immunofluorescent staining was performed using anti-Strep-Tag II antibody. DAPI was used for the nucleus. Three independent experiments were performed and the representative results are shown here. Scale bar, 20 μm.

RUNX2 contains several functional domains. Citrullinated R25 and R26 of mRUNX2 were located in the activation domain, six sites (R188, R193, R197, R232, R235, and R236) were within the Runt domain, and R381 and R393 were within the PST domain (Supplementary Fig. 11). To investigate which sites play a key role in the maintenance of RUNX2 protein stability, MC3T3-E1 cells transfected with *Strep-mRunx2* wild-type (WT) or mutant forms were cultivated in osteogenic media, and RUNX2 levels were analyzed by western blotting. Interestingly, mRUNX2 R381K mutation among the ten R to K mutations dramatically decreased RUNX2 levels compared to RUNX2 WT (Fig. 6A). To further validate the effect of R381K mutation on RUNX2 stability, WT or R381K mutant constructs were introduced into pOB cells and hMSCs. Western blot analysis showed that mutation in the mRUNX2 R381 locus also greatly reduced RUNX2 levels in both cells (Fig. 6B, C). Core binding factor β (Cbfβ) conditional knockout mice have shown that Cbfβ is required for osteoblast differentiation [22, 33, 34]. Previous studies have demonstrated that Cbfβ is important in the stabilization of RUNX2 by protecting it from degradation by ubiquitination [22, 34]. Cbfβ interacts with the Runt domain that is highly conserved in the RUNX family proteins [35]. Because six sites (R188, R193, R197, R232, R235, and R236) citrullinated by PADI2 were located within the Runt domain of mRUNX2 (Fig. 5C; Supplementary Fig. 11), we investigated whether the ten R mutations, including these six R sites, affected the heterodimerization of RUNX2 with Cbfβ. 293 T cells were transfected with *Strep-mRunx2* WT or mutant forms with or without *Myc-Cbfβ*. Since the expression levels of RUNX2 variants were slightly different, co-immunoprecipitation (Co-IP) was performed after adjusting the expression level of Strep-RUNX2 proteins similarly. Co-IP experiments showed that none of the RUNX2 mutations suppressed the dimerization of RUNX2 with Cbfβ (Fig. 6D). RUNX2 R25K, R26K, and R236K appeared to interact more strongly with Cbfβ than the WT, but this was thought to be due to the higher expression levels of Strep-RUNX2 and Myc-Cbfβ in the Co-IP samples of these mutant groups than in the WT control group. Next, we examined whether these mutations affected the nuclear localization of RUNX2, which functions as a transcription factor. Mutations located near the nuclear localization signal (NLS) (R232K, R235K, and R236K) or near the nuclear matrix target signal (NMTS) (R381K and R393K) did not significantly affect the nuclear localization of RUNX2 (Fig. 6E). The other remaining RUNX2 mutations also did not affect the nuclear localization of the RUNX2 protein (Supplementary Fig. 12). Taken together, these results demonstrate that the citrullination of R381 of RUNX2 plays a critical role in maintaining RUNX2 stability. However, these mutations did not significantly affect the binding of RUNX2 to Cbfβ or the nuclear accumulation of RUNX2.

## Discussion

In the field of bone biology, citrullination has received less attention compared to other post-translational modifications. However, in autoimmune diseases such as rheumatoid arthritis, it is known that overactivation of PADIs can lead to citrullination of specific proteins like fibrinogen and vimentin, triggering an immune response that contributes to the disease [36, 37]. While previous studies have highlighted the association of PADI overexpression or hyperactivity with pathophysiology, little is known about the role of PADIs and protein citrullination in normal physiological bone tissue. In our study, we demonstrated that PADI2 plays a crucial role in osteoblast differentiation and the communication between osteoblasts and osteoclasts, which is vital for maintaining bone homeostasis. We also observed that PADI2 deficiency leads to bone loss and a human CCD-like phenotype. Moreover, we discovered that PADI2-mediated citrullination of RUNX2, an essential transcription factor that regulates osteoblast differentiation and function, contributes to the stabilization of RUNX2.

Here, *EIIA-Cre*-mediated global *Padi2* KO mice exhibited reduced osteoblast differentiation and increased osteoclastogenesis, which resulted in reduced bone mass. *Padi2*-deficient BMMs are accelerated into multinucleated mature osteoclasts in these global *Padi2* KO mice. These results suggest that *Padi2* deficiency in osteoblasts reduces osteoblast function, but its deficiency in BMMs promotes differentiation into osteoclasts, resulting in reduced bone formation, accelerated bone destruction, and ultimately increased bone loss. Osteoblast-specific *Padi2* deletion using *Col1α1(2.3* *kb)-Cre* transgenic mice also increased TRAP-positive osteoclasts compared to control mice, indicating that soluble factors secreted from *Padi2*-deficient osteoblasts are also involved in promoting osteoclastogenesis. Our data showed a significant increase in representative osteoclastogenic factors *Csf-1* and *Rankl* mRNA levels in *Padi2* KO pOB cells compared to control cells. However, in our data, RUNX2, known as a key transcription factor regulating the expression of these genes [38, 39], was greatly reduced when *Padi2* was deficient. Therefore, the increase in *Csf-1* and *Rankl* transcripts in *Padi2*-deficient pOBs was possibly promoted by other transcription factors activated by *Padi2* depletion. NF-κB p65 transcription factor is one of the candidates involved in this regulation. NF-κB p65 is activated by *Padi2* knockdown in osteoblasts [8] and NF-κB p65 binds to the M-CSF promoter in myeloid cell lines [40]. Also, NF-κB signaling pathway mediates HGF-promoted RANKL expression in osteoblasts and bone marrow stromal cells [41]. In addition to these genes, NF-κB promotes the expression and secretion of the senescence-associated secretory phenotype (SASP) factors CCL2, CCL5, and CCL7 in *Padi2*-deficient osteoblasts [8], which can promote the recruitment of monocytes and osteoclastogenesis [42–44]. The inhibition of NF-κB signaling pathway using a pharmacological inhibitor or RNAi significantly reduced the upregulated levels of these genes by *Padi2* knockdown in osteoblasts [8], suggesting that blocking NF-κB signaling pathway can be a therapeutic target that can reduce the abnormally increased osteoclastogenic factors and restore the function of osteoblasts lost by reduced PADI2.

The PADI family of enzymes consists of five isozymes (PADI1-4 and PADI6), which exhibit unique tissue localization and have overlapping substrate specificities. These isozymes show high homology among both orthologs and paralogs, with amino acid identities ranging from 44% to 58% among human PADI paralogs [45, 46]. Among these isozymes, PADI2 is the predominantly expressed isozyme in mesenchymal cell-derived osteoblasts, and its loss accelerates cellular senescence and severely inhibits osteoblast differentiation, suggesting that PADI2 plays an important role in osteoblast differentiation and function [8]. However, contrary to expectations, severe developmental bone defects were not observed in these mice although *Padi2* KO mice displayed a CCD phenotype and bone loss. The mRNA levels of other PADI isozymes, which were suspected to compensate for the loss of PADI2, were actually lower in *Padi2* KO pOB cells compared to control cells. This indicates that the bone phenotype observed in *Padi2* KO mice was not due to compensation by other isozymes. Furthermore, our previous study showed that the expression of PADI2 was low during osteoblast proliferation but increased as differentiation progresses, and loss of PADI2 due to oxidative stress has been shown to induce DNA damage and the SASP factors, leading to osteoblast senescence [8]. These findings suggest that PADI2 is important for defending against aging-associated oxidative stress and maintaining cellular homeostasis. Therefore, it is believed that PADI2 may play a more significant role in maintaining bone homeostasis in mature or aged bone rather than during early bone development. Collectively, our previous and current studies highlight the importance of PADI2 in osteoblast differentiation, bone homeostasis, and defense against oxidative stress. Thus, its deficiency leads to bone loss and compromises the maintenance of bone integrity, particularly in mature or aged bone.

RUNX2 is regulated by various post-translational modifications. The fibroblast growth factor (FGF)/FGF receptor (FGFR) and bone morphogenetic protein (BMP)/BMP receptor (BMPR) signaling pathways, which are essential for osteoblast proliferation and differentiation, induce the acetylation and phosphorylation of RUNX2, which enhances its stability and transcriptional activity [18, 19]. Furthermore, the acetylation of RUNX2 by FGF2 requires the phosphorylation of RUNX2 by ERK MAPK and the subsequent isomerization of RUNX2 by PIN1, which recognizes the phosphorylation of RUNX2 [47]. However, the role and regulatory mechanism of PADI enzyme-mediated citrullination in osteoblast differentiation and function have not been extensively studied until now. Also, the involvement of citrullination in regulating key factors of osteoblast differentiation, such as RUNX2, has not been reported. This study provides novel insights by demonstrating that PADI2 is involved in protecting RUNX2 from ubiquitin-mediated proteasomal degradation. The findings suggest that the citrullination of RUNX2 by PADI2 plays a crucial role in maintaining the stability of the RUNX2 protein. However, it is important to note that the citrullination of RUNX2 does not act alone but likely cooperates with other PTMs to regulate RUNX2 stability. This is supported by the partial rescue of RUNX2 protein levels observed with MG132 treatment in *Padi2* knockout and knockdown cells. Furthermore, citrullination at the R381 site of RUNX2 has been identified as important for maintaining the stability of the RUNX2 protein. This suggests that the citrullination of this particular site is critical for the regulatory function of PADI2 on RUNX2 stability.

Contrary to expectations, site-directed mutagenesis of the citrullination sites identified in this study did not inhibit the heterodimerization of RUNX2 with Cbfβ nor did it affect the nuclear localization of RUNX2. However, although not revealed in this study, citrullination of these R sites in RUNX2 can affect various functions of RUNX2, such as protein-protein interactions, DNA-binding activity, and transcriptional activity. In addition, citrullination of RUNX2 may induce conformational changes in RUNX2, allowing it to better bind to the promoter region of target genes or proteins involved in regulating RUNX2 stability. Further studies are required to answer these questions.

In this study, *Padi2* KO mice exhibited a human CCD-like phenotype. CCD is usually caused by haploinsufficiency of RUNX2 due to mutations in humans [48]. Mass spectrometry revealed that PADI2 directly citrullinated 10 R sites within the RUNX2 protein. These results indicate that post-translational citrullination of RUNX2 by PADI2 can modulate RUNX2 function, and its dysregulation can lead to bone diseases, such as CCD. Among the 10 R citrullination sites of RUNX2 identified in this study, missense mutations at sites corresponding to R193, R197, R232, and R381 of mRUNX2 isoform 1 have been reported in human patients with CCD [48–53]. In approximately 30 percent of the individuals with CCD, no mutations in the *RUNX2* gene have been found. The cause of this condition remains unclear. However, these patients may have a loss-of-functional mutation of the *PADI2* gene, which in turn affects RUNX2 function. Here, we show that citrullination of RUNX2 at R381 by PADI2 is an essential post-translational modification for maintaining RUNX2 protein stability. Although a direct correlation between citrullination and the R-site mutations within RUNX2 reported in patients with CCD has not been elucidated, this strongly suggests that citrullination at these R sites may play an important role in RUNX2 function. Taken together, these results suggest that citrullination of RUNX2 is a critical post-translational modification for osteoblast differentiation and function.

Collectively, we highlighted, for the first time, the critical role of PADI2 in bone formation and homeostasis using global and osteoblast-specific conditional *Padi2* KO mice, shedding light on its underlying mechanisms. *Padi2* deficiency leads to a reduction in bone mass and the development of the CCD phenotype, primarily due to decreased stability of the RUNX2 protein. In vitro mechanistic analyses have demonstrated that PADI2 citrullinates RUNX2 and prevents its proteasomal degradation. This study provides novel evidence elucidating the involvement of PADI2 and its citrullination in osteoblast differentiation and function, thereby opening up new avenues for targeting bone diseases, including CCD and senile osteoporosis, in potential therapeutic interventions

### Reporting summary

Further information on research design is available in the Nature Research Reporting Summary linked to this article.

## Supplementary information

Supplementary Information

Original Data File

Reporting Summary

## Data Availability

Data supporting the findings of this study are available from the corresponding author upon request. The authors follow the guidelines provided by the journal for sharing the data. Mass spectrometry proteomics data are available via ProteomeXchange with the identifier PXD040179.
