# Supplementary material for: Peptidylarginine deiminase 2 plays a key role in osteogenesis by enhancing RUNX2 stability through citrullination
Source: Cell Death Dis. 2023 Aug 30;14(8):576. doi: 10.1038/s41419-023-06101-7 (PMC10468518; doi:10.1038/s41419-023-06101-7)
Supplement: Supplementary file 1 — Supplementary Information [file 41419_2023_6101_MOESM1_ESM.docx]

**Supplementary Information**

**Peptidylarginine deiminase 2 plays a key role in osteogenesis by enhancing RUNX2 stability through citrullination**

Hyun-Jung Kim^1,#^, Hye-Rim Shin^1, #^, Heein Yoon^1^, Min-Sang Park^1^, Byung-Gyu Kim^2^, Jae-I Moon^1^, Woo-Jin Kim^1^, Seung Gwa Park^1^, Ki-Tae Kim^1^, Ha-Neui Kim^3^, Je-Yong Choi^4^, Hyun-Mo Ryoo^1,^*

^1^Department of Molecular Genetics and Dental Pharmacology, School of Dentistry and Dental Research Institute, Seoul National University, Seoul, South Korea

^2^Center for Genomic Integrity, Institute for Basic Science (IBS), Ulsan, South Korea

^3^Center for Musculoskeletal Disease Research and Center for Osteoporosis and Metabolic Bone Diseases, Department of Internal Medicine, Division of Endocrinology and Metabolism, University of Arkansas for Medical Sciences, Little Rock, Arkansas, USA

^4^Department of Biochemistry and Cell Biology, Cell and Matrix Research Institute, BK21 Plus KNU Biomedical Convergence Program, Skeletal Disease Analysis Center, Korea Mouse Phenotyping Center, School of Medicine, Kyungpook National University, Daegu, South Korea

**^#^** These authors have equally contributed to this work as co-first authors

*Corresponding Author: Hyun-Mo Ryoo, Department of Molecular Genetics & Dental Pharmacology, School of Dentistry, Seoul National University, 1 Gwanak-ro, Gwanak-gu, Seoul, 08826, South Korea

Email: hmryoo@snu.ac.kr

**
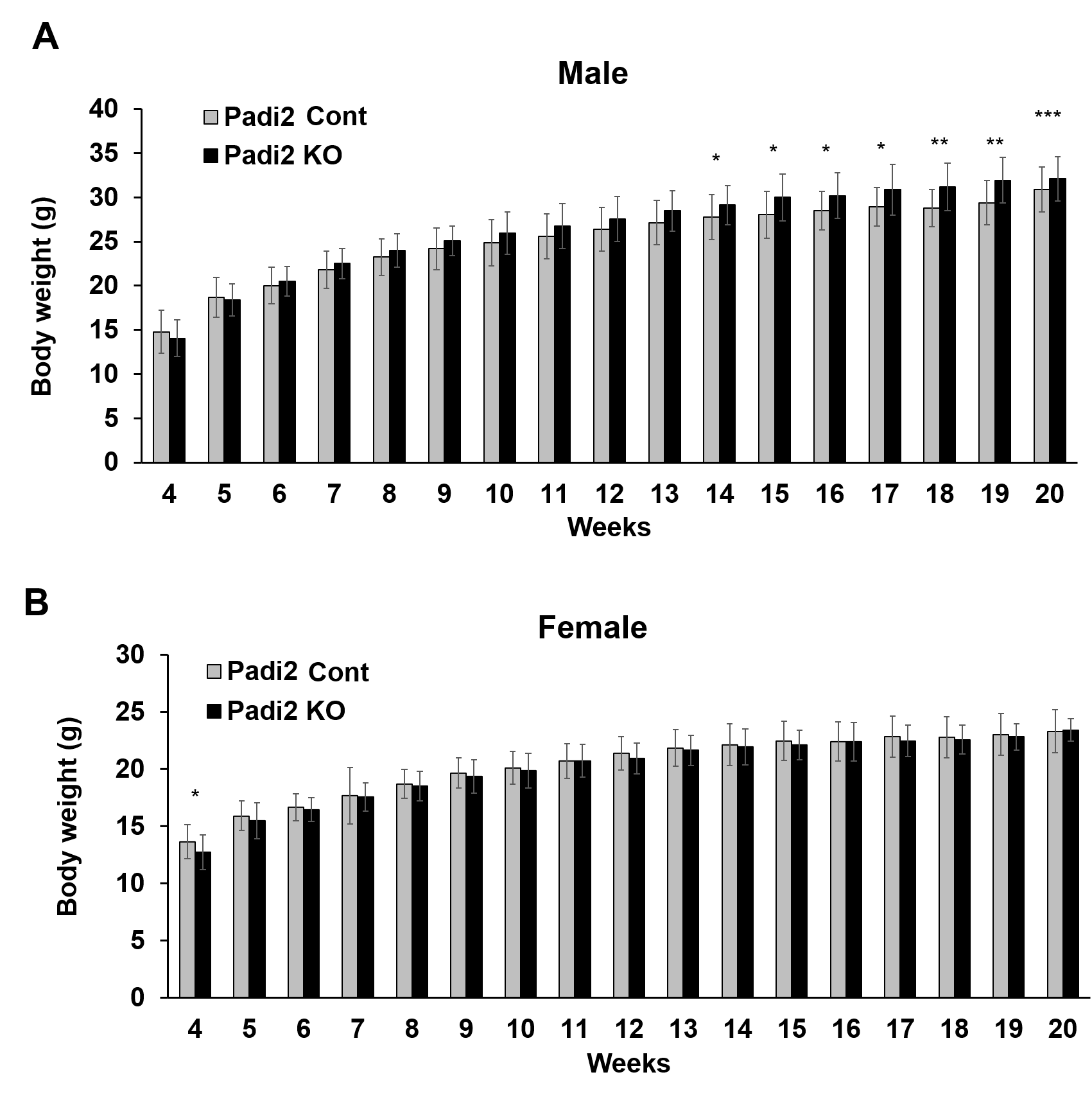
**

**Supplementary Fig. 1 Body weight was not big different between *Padi2* Cont and KO mice. A, B** Body weights of male **(A)** and female **(B)** *Padi2* Cont and KO mice were measured weekly from 4 weeks-age to 20-weeks age (n = 9-32 in each group). Results are shown as mean±SD (n = 9-32 in each group). *P<0.05, **P<0.01, ***P<0.001.

**
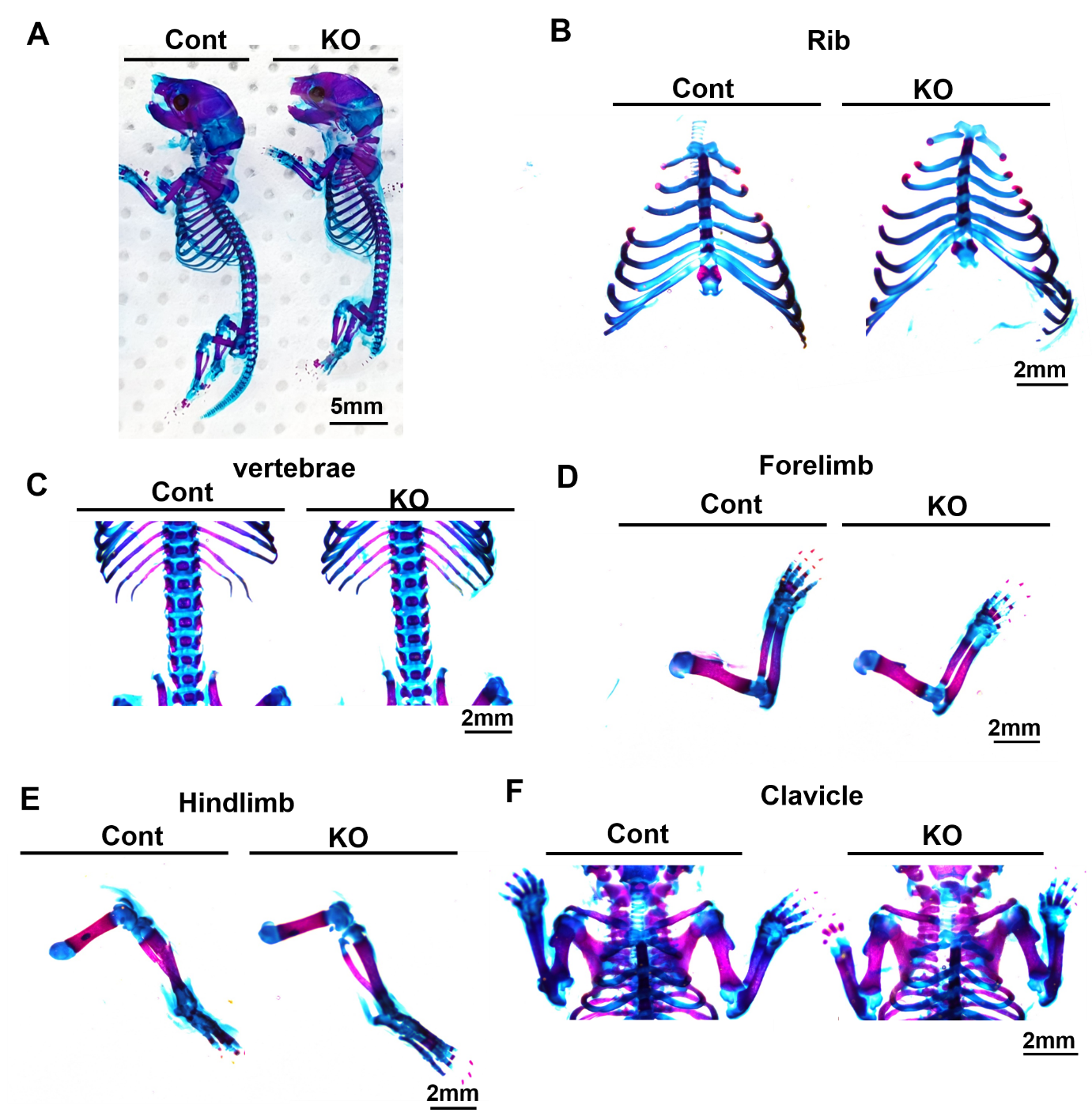
**

**Supplementary Fig. 2 Newborn *Padi2* KO mice showed mild bone hypoplasia. A-F** Whole-mount skeleton staining of *Padi2* Cont (n=6) and KO (n=5) newborn littermates by Alizarin red and Alcian blue staining. Scale bar: 5mm, whole body and 2mm, others.

**
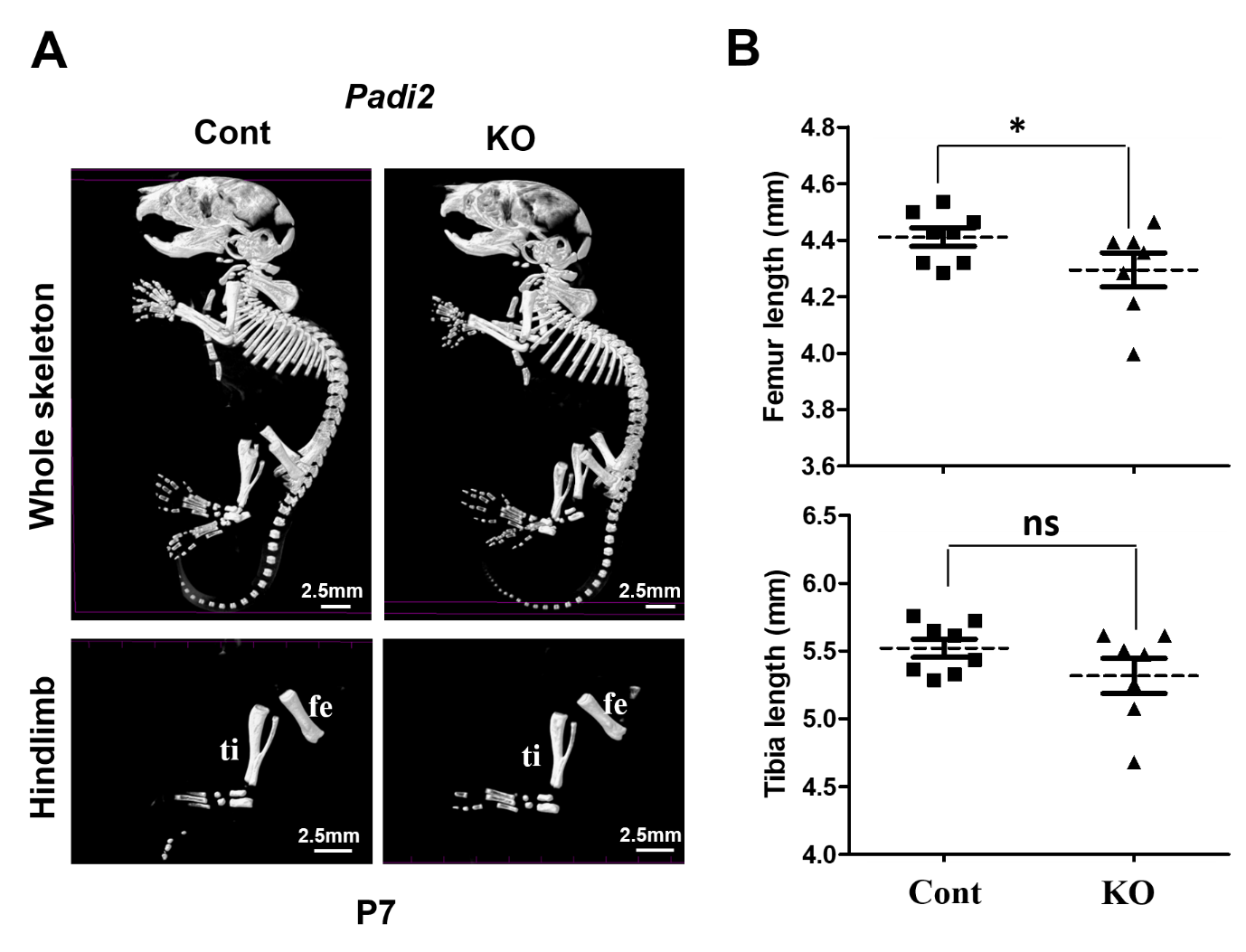
**

**Supplementary Fig. 3 *Padi2* KO mice showed reduced cranial ossification and femur length compared to Cont littermates at P7. A** Representative micro-CT images of whole skeleton (upper panel) and hindlimb (lower panel) of P7-old *Padi2* Cont (n=8) and KO (n=7) mice. **B** Femur length (upper) and tibia length of P7-old *Padi2* Cont and KO mice. *P<0.05, ns, not significant.

**
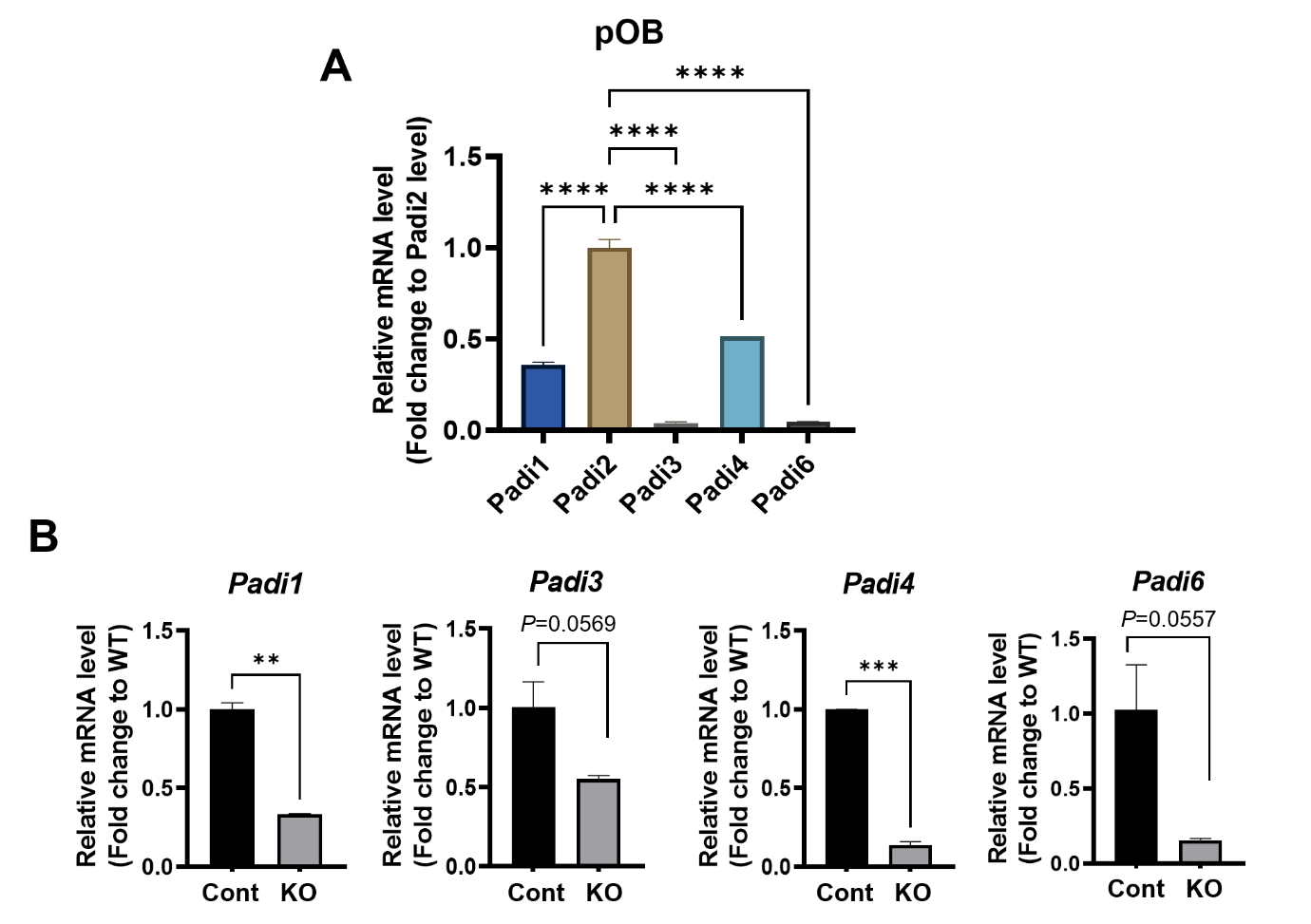
**

**Supplementary Fig. 4 *Padi2* KO deficiency did not cause compensation of other PADI isozymes. A** Relative mRNA expression levels of *Padi1, 2, 3, 4, and 6* in WT primary calvaria OBs cultured in osteogenic medium for 4 days were determined by RT-qPCR. Three independent experiments with three biological replicates for each group. **B** Relative mRNA expression levels of *Padi1, 2, 3, 4, and 6* in *Padi2* Cont and KO primary calvarial OBs, which were cultured in osteogenic medium for 4 days, were determined by RT-qPCR. Three independent experiments with three biological replicates for each group. Data are expressed as the mean± SD. **P<0.01, ***P<0.001, ****P<0.0001.

**
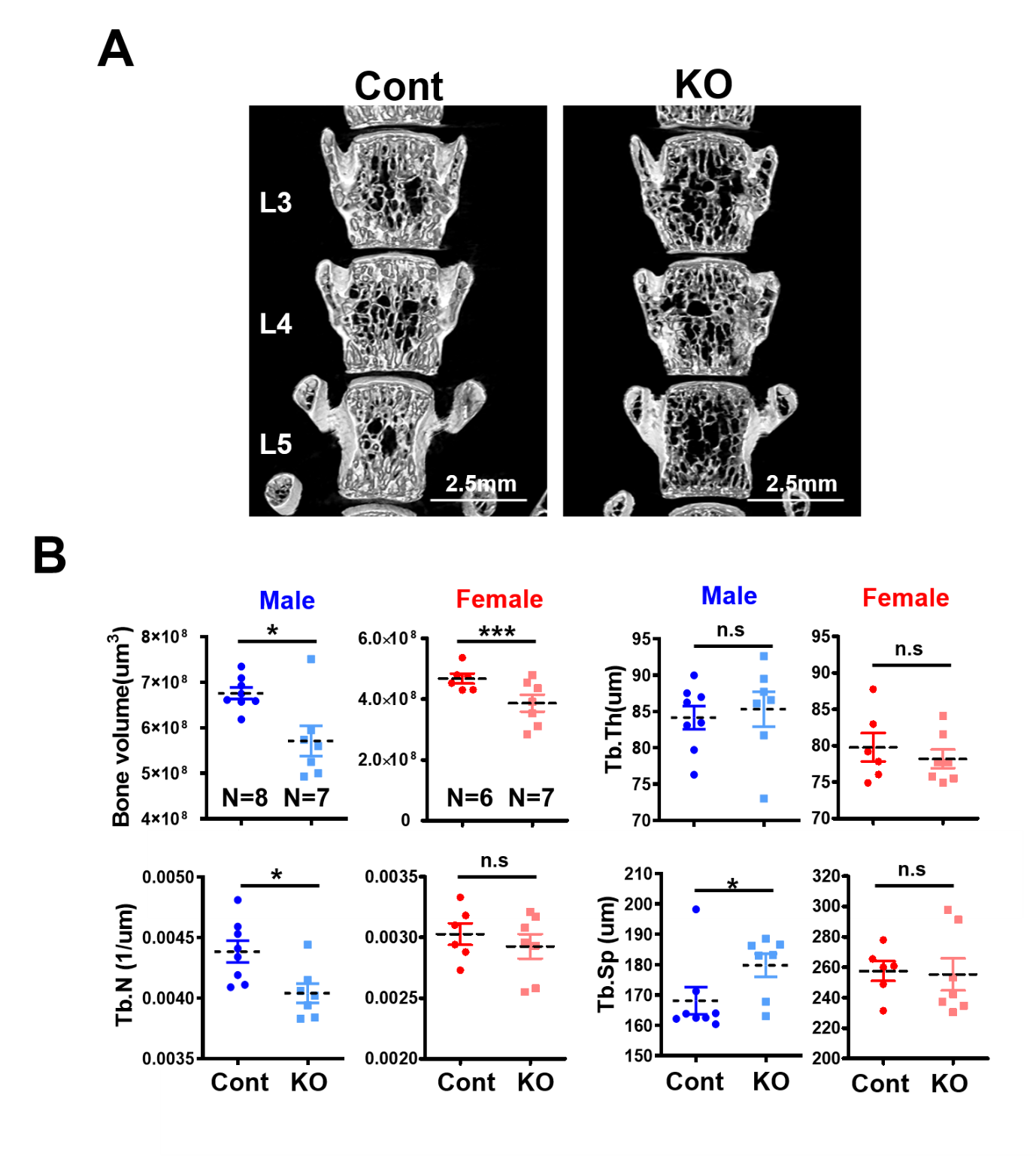
Supplementary Fig. 5 Vertebral bone volume was significantly reduced in 4-month-old *Padi2* KO mice relative to Cont mice. A, B** Representative micro-CT images of L3-L5 vertebral body **(A)** and micro-CT analysis of L4 vertebral bone **(B)** of 4-month-old *Padi2* Cont and KO mice in both male (n=8, Cont mice and n=7, Padi2 KO mice) and female (n=6, Cont mice and n=7, *Padi2* KO mice). Tb.Th, trabecular thickness; Tb.N, trabecular number; Tb.Sp, trabecular separation. *P<0.05, **P<0.01, ***P<0.001, ns, not significant.

**
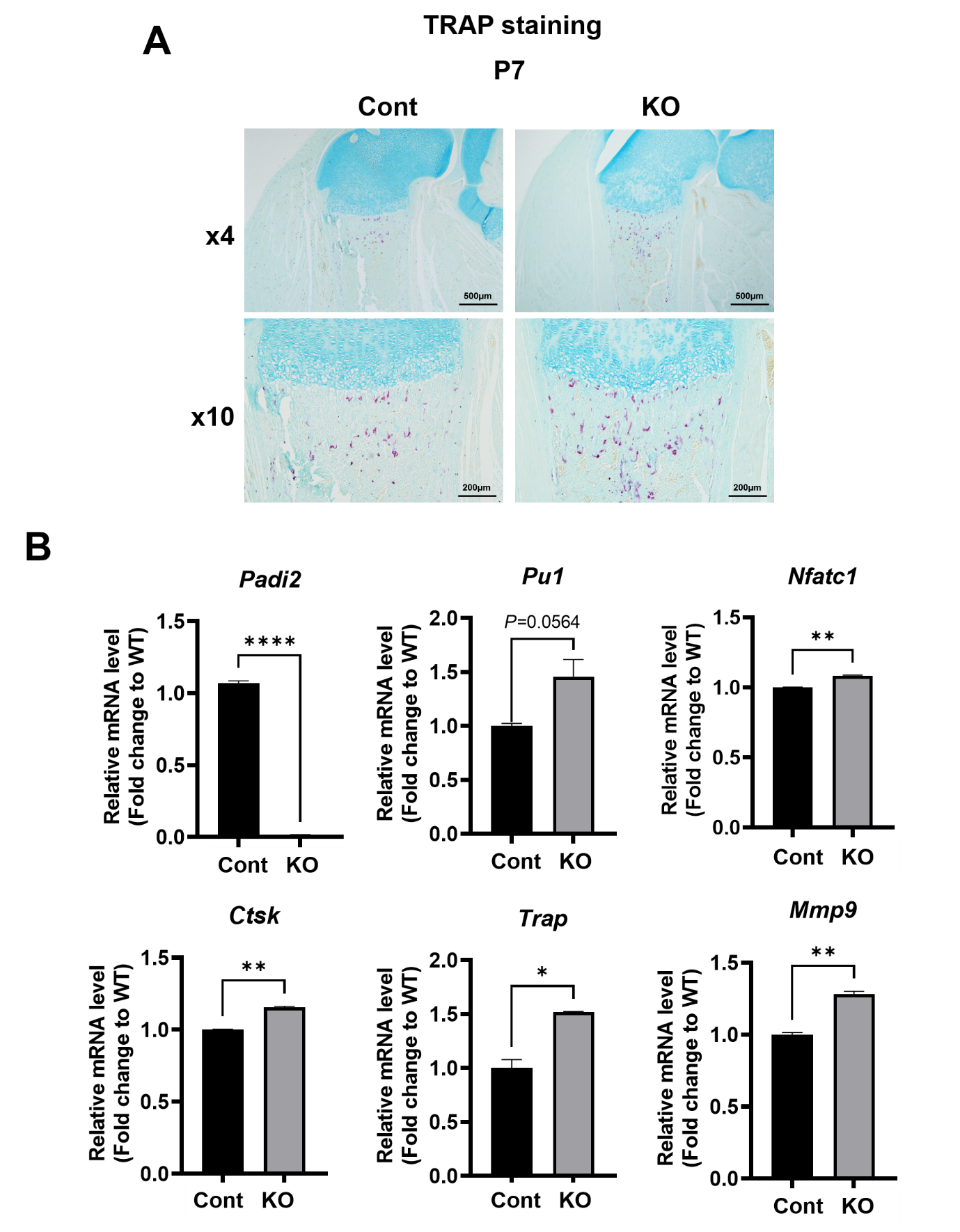
**

**Supplementary Fig. 6 Padi2 deficiency increased osteoclastogenesis. A** Representative images of TRAP-stained trabecular bone of distal femur from P7-old *Padi2* Cont and KO mice. TRAP-positive purple spots indicate multinucleated osteoclasts. Three independent experiments with three biological replicates for each group. Scale bar: 500μm (upper panel), 200μm (lower panel). **B** Relative mRNA expression levels of *Padi2* and osteoclast differentiation markers including *Pu1*, *Nfatc1*, *Ctsk*, *Trap*, and *Mmp9* were confirmed in *Padi2* Cont and KO osteoclasts by RT-qPCR. Three independent experiments with three biological replicates for each group. Data are expressed as the mean± SD. ***P<0.001. ND, not determined.

**
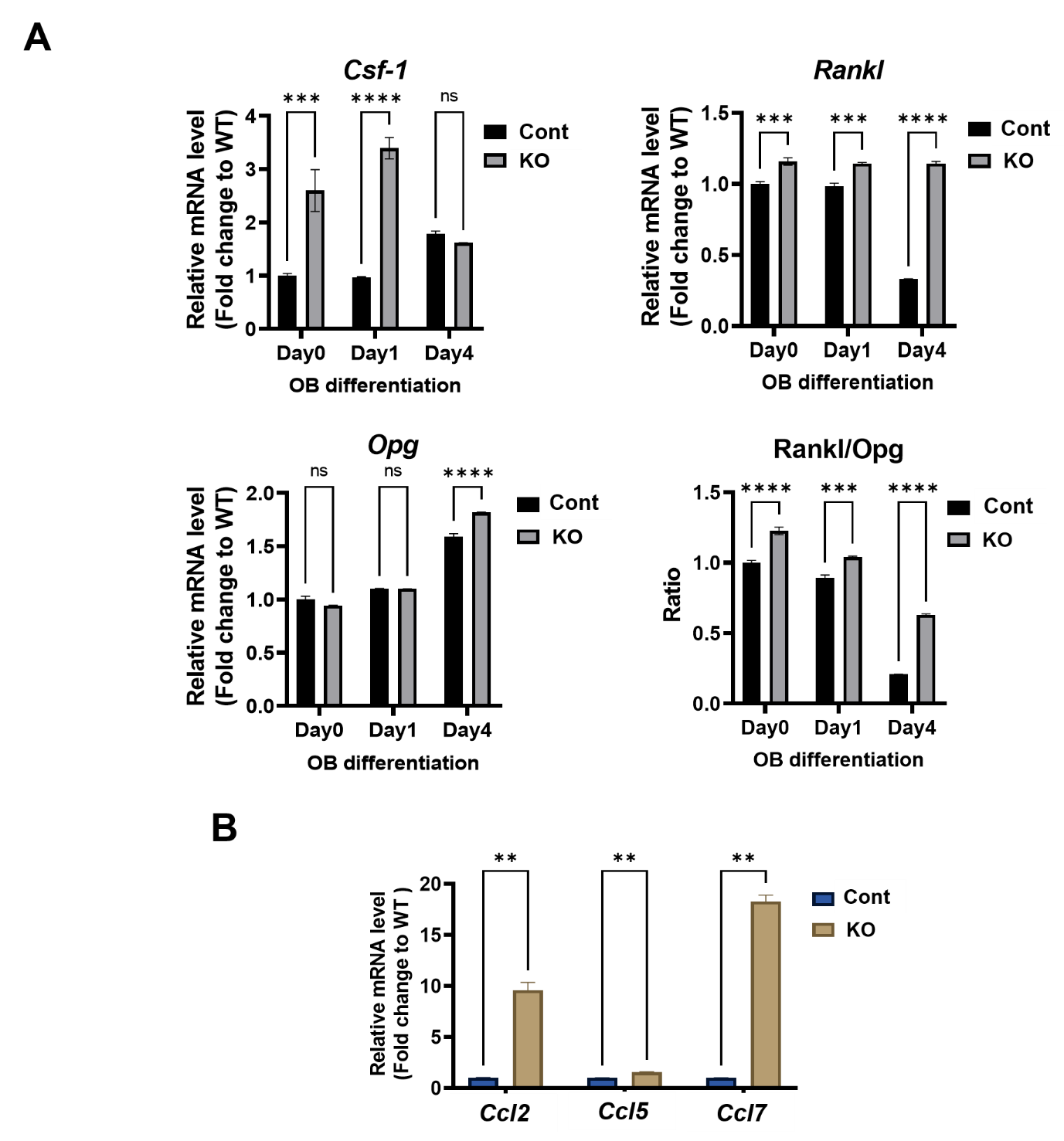
**

**Supplementary Fig. 7 Osteoclastogenic factors increased in *Padi2* KO pOBs compared to Cont pOBs. A** Relative mRNA expression levels of *Csf-1, Rankl* and *Opg* in *Padi2* Cont and KO pOBs cultured or not (Day 0) in osteogenic medium for each indicated day were determined by RT-qPCR and the relative ratio of Rankl/Opg was shown. Three independent experiments with three biological replicates for each group. **B** Relative mRNA expression levels of *Ccl2, Ccl5* and *Ccl7* in *Padi2* Cont and KO pOBs cultured in osteogenic medium for 4 days were determined by RT-qPCR. Three independent experiments with three biological replicates for each group. Data are expressed as the mean± SD. **P<0.01, ***P<0.001, ****P<0.0001. ns, not significant.


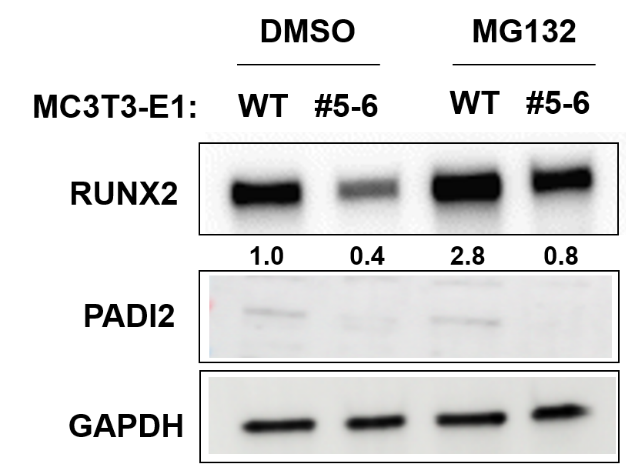


**Supplementary Fig. 8 PADI2 protected RUNX2 from ubiquitin-proteasomal degradation.** CRISPR–Cas9-mediated *Padi2* KO cell clone #5-6 and control cells were cultured in osteogenic media for 2 days, and 20 μM MG132 or DMSO as vehicle was treated for 6 hours before harvesting cells, and western blot analysis followed. GAPDH was used as a loading control. RUNX2 level was quantified using ImageJ software and normalized with GAPDH. Western blot data were collected from at least two or three independent experiments; the representative results are shown here.

**
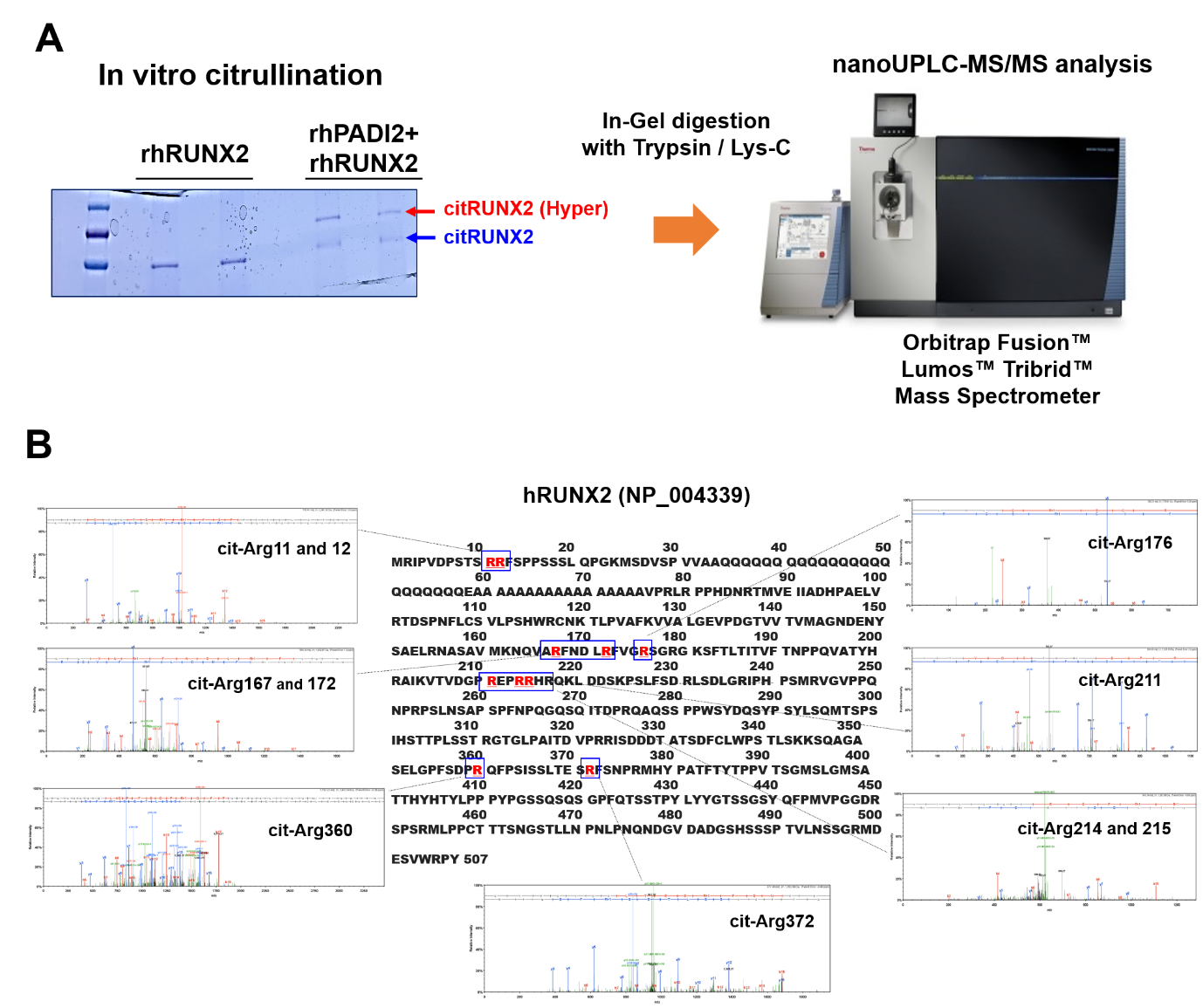
**

**Supplementary Fig. 9 Identification of PADI2-mediated citrullination sites within RUNX2 via LC-MS/MS analysis. A** Schematic representation of the method used for identification of RUNX2 citrullination sites. rhRUNX2 isoform c protein was *in vitro* citrullinated with or without rhPADI2, followed by separation by SDS-PAGE and Coomassie staining. After destaining, bands were cut and digested with trypsin followed by the performing of LC-MS/MS analysis. **B** Identification of 10 citrullinated peptides containing R11, R12, R167, R172, R176, R211, R214, R215, R360, and R372 within hRUNX2 isoform c (NP_004339).


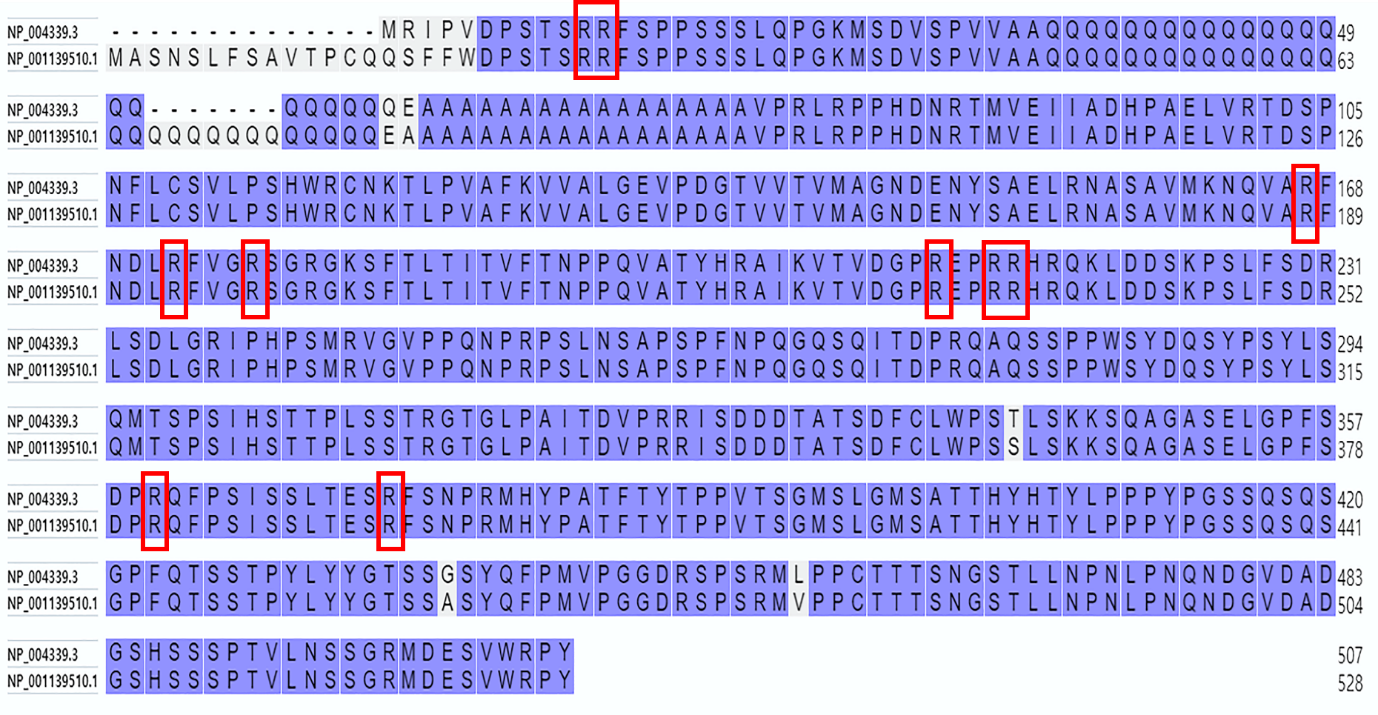
**Supplementary Fig. 10 Homology comparison of hRUNX2 isoform c (NP_004339) and mRUNX2 isoform 1 (NP_001139510).** Two protein sequences were aligned using UniProt alignment tool (https://www.uniprot.org/align). Red squares indicate arginine sites citrullinated by PADI2.

**
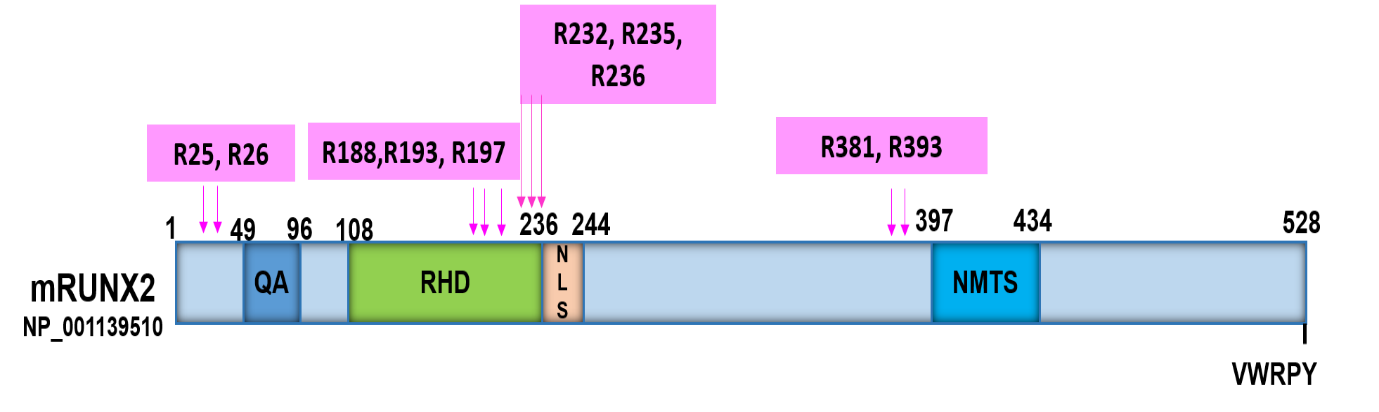
**

**Supplementary Fig. 11 The functional domains of mouse RUNX2 isoform 1 (bone specific isoform) (NP_001139510) and PADI2-mediated 10 citrullination sites (R25, R26, R188, R193, R197, R232, R235, R236, R381, and R393) matching those of hRUNX2 isoform c.**

**
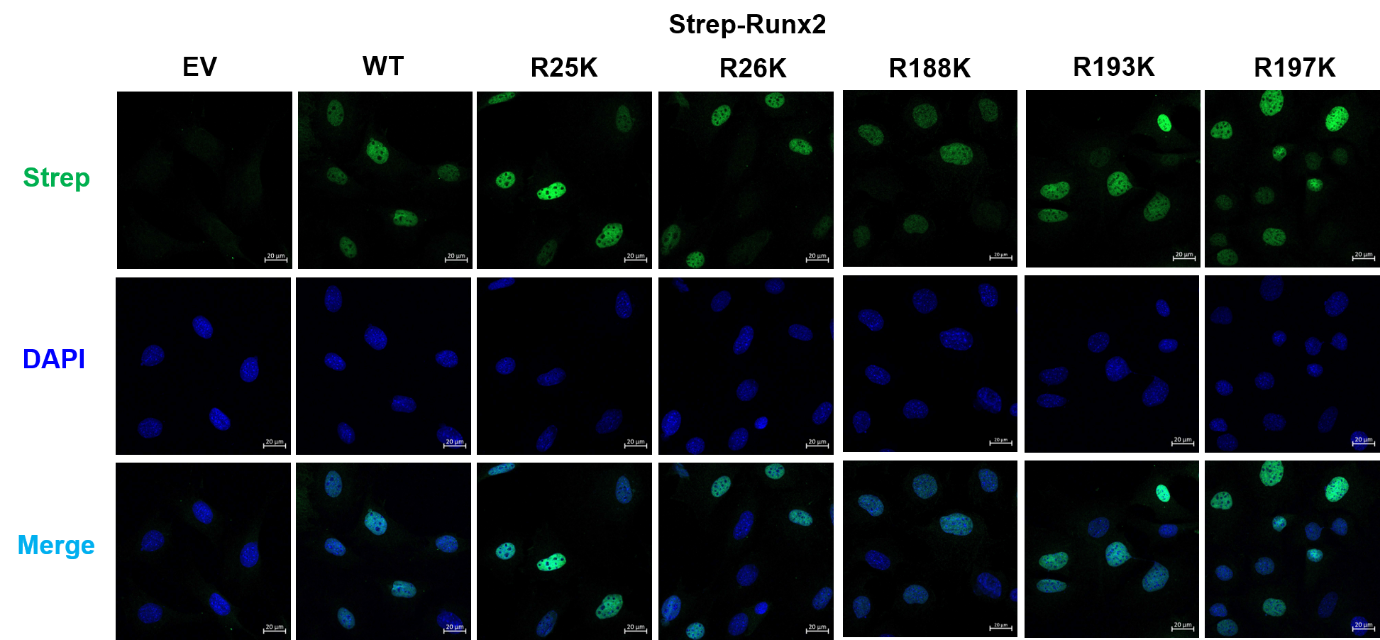
**

**Supplementary Fig. 12 PADI2-mediated citrullination of RUNX2 did not affect its nuclear localization.** MC3T3-E1 cells were transfected with Strep-Runx2 wild type (WT) or R-to-K mutants (R25K, R26K, R188K, R193K, and R197K) and cultured for 2 days after transfection. Cells were fixed with 4% PFA, permeabilized, and then immunofluorescent staining was performed using anti-Strep-Tag II antibody. DAPI was used for nucleus. Three independent experiments were performed and the representative results are shown here. Scale bar, 20 μm.

**Supplementary Table 1. Primer sequences used for RT-qPCR analysis in this study (forward, FOR; reverse, REV)**

| **Gene** | **Sequences for primers** |
| --- | --- |
| ***Padi1*** | FOR: CGTGCAGAAATGCATCGACT |
|  | REV: ATGTCCACGATGTCGCTCTC |
| ***Padi2*** | FOR: GACAAGGTCACTGTCAACTACTATGAA |
|  | REV: TTGTTCTTCTCCACCTCTCCAT |
| ***Padi3*** | FOR: CCTAGGCCGGCATGTCTCTA |
|  | REV: CTCAGGAACCGCCCCATAAA |
| ***Padi4*** | FOR: GGCTACACAACCTTCGGCAT |
|  | REV: GCTGCTTTCACCTGTAGGGT |
| ***Padi6*** | FOR: GTGGCTAGCTTGGTAAGCCC |
|  | REV: TGCACACTTGCTGATGTCCAA |
| ***Runx2*** | FOR: TTCTCCAACCCACGAATGCAC |
|  | REV: CAGGTACGTGTGGTAGTGAGT |
| ***Alp*** | FOR: CCAACTCTTTTGTGCCAGAGA |
|  | REV: GGCTACATTGGTGTTGAGCTTTT |
| ***Ocn*** | FOR: CTGACAAAGCCTTCATGTCCAA |
|  | REV: GCGCCGGAGTCTGTTCACTA |
| ***Bsp*** | FOR: CAGGGAGGCAGTGACTCTTC |
|  | REV: AGTGTGGAAAGTGTGGCGTT |
| ***Padi2* for Padi2 cKO** | FOR: GTGGAGGTCGTACGTGATGG |
|  | REV: ATCTCAATGGCCGTGAGGAA |
| ***Csf-1*** | FOR: GACCCTCGAGTCAACAGAGC |
|  | REV: TGTCAGTCTCTGCCTGGATG |
| ***Rankl*** | FOR: CAGCCATTTGCACACCTCACCAT |
|  | REV: TTTCGTGCTCCCTCCTTTCATCAG |
| ***Opg*** | FOR: ACCCAGAAACTGGTCATCAGC |
|  | REV: CTGCAATACACACACTCATCACT |
| ***PU1*** | FOR: AGAAGCTGATGGCTTGGAGC |
|  | REV: GCGAATCTTTTTCTTGCTGCC |
| ***Mmp9*** | FOR: CTGGACAGCCAGACACTAAAG |
|  | REV: CTCGCGGCAAGTCTTCAGAG |
| ***Nfatc1*** | FOR: GGTGCCTTTTGCGAGCAGTATC |
|  | REV: CGTATGGACCAGAATGTGACGG |
| ***Ccl2*** | FOR: TGCTGACCCCAAGAAGGAAT |
|  | REV: GAAGTGCTTGAGGTGGTTGTG |
| ***Ccl5*** | FOR: AGATCTCTGCAGCTGCCCTCA |
|  | REV: GGAGCACTTGCTGCTGGTGTAG |
| ***Ccl7*** | FOR: CTTTCAGCATCCAAGTGTGGG |
|  | REV: ATGCTATAGCCTCCTCGACC |
| ***Gapdh*** | FOR: GGCCTCACCCCATTTGATGT |
|  | REV: CATGTTCCAGTATGACTCCACTC |

**Supplementary Table 2. Oligonucleotide sequences used for Arg (R) to Lys (K) site-directed mutagenesis of mouse Runx2 transcript 1 citrullination sites (forward, FOR; reverse, REV)**

| **Gene** | **Sequences for primers (5’ to 3’)** |
| --- | --- |
| **Runx2-R25K** | Original seq: GATCCGAGCACCAGC**CGG**CGCTTCAGCCCCCCCT |
|  | FOR: GATCCGAGCACCAGC**AAG**CGCTTCAGCCCCCCCT |
|  | REV: AGGGGGGGCTGAAGCG**CTT**GCTGGTGCTCGGATC |
| **Runx2-R26K** | Original seq: GAGCACCAGCCGG**CGC**TTCAGCCCCCCCT |
|  | FOR: GAGCACCAGCCGG**AAG**TTCAGCCCCCCCT |
|  | REV: AGGGGGGGCTGAA**CTT**CCGGCTGGTGCTC |
| **Runx2-R193K** | Original seq: AGGTTCAACGATCTG**AGA**TTTGTGGGCCGGAG |
|  | FOR: AGGTTCAACGATCTG**AAG**TTTGTGGGCCGGAG |
|  | REV: CTCCGGCCCACAAA**CTT**CAGATCGTTGAACCT |
| **Runx2-R197K** | Original seq: CTGAGATTTGTGGGC**CGG**AGCGGACGAGGCAAGA |
|  | FOR: CTGAGATTTGTGGGC**AAG**AGCGGACGAGGCAAGA |
|  | REV: TCTTGCCTCGTCCGCT**CTT**GCCCACAAATCTCAG |
| **Runx2-R232K** | Original seq: ACAGTGGACGGTCCC**CGG**GAACCAAGAAGGCACA |
|  | FOR: ACAGTGGACGGTCCC**AAG**GAACCAAGAAGGCACA |
|  | REV: TGTGCCTTCTTGGTTC**CTT**GGGACCGTCCACTGT |
| **Runx2-R235K** | Original seq: GTCCCCGGGAACCA**AGA**AGGCACAGACAGAAG |
|  | FOR: GTCCCCGGGAACCA**AAG**AGGCACAGACAGAAG |
|  | REV: CTTCTGTCTGTGCCT**CTT**TGGTTCCCGGGGAC |
| **Runx2-R236K** | Original seq: CCGGGAACCAAGA**AGG**CACAGACAGAAG |
|  | FOR: CCGGGAACCAAGA**AAG**CACAGACAGAAG |
|  | REV: CTTCTGTCTGTG**CTT**TCTTGGTTCCCGG |
| **Runx2-R381K** | Original seq: CTTTTTCAGACCCC**AGG**CAGTTCCCAAGCATT |
|  | FOR: CTTTTTCAGACCCC**AAG**CAGTTCCCAAGCATT |
|  | REV: AATGCTTGGGAACTG**CTT**GGGGTCTGAAAAAG |
| **Runx2-R393K** | Original seq: TCCCTCACTGAGAGC**CGC**TTCTCCAACCCACGAA |
|  | FOR: TCCCTCACTGAGAGC**AAG**TTCTCCAACCCACGAA |
|  | REV: TTCGTGGGTTGGAGAA**CTT**GCTCTCAGTGAGGGA |
