# Supplementary material for: Peptidylarginine deiminase 2 plays a key role in osteogenesis by enhancing RUNX2 stability through citrullination
Source: Cell Death Dis. 2023 Aug 30;14(8):576. doi: 10.1038/s41419-023-06101-7 (PMC10468518; doi:10.1038/s41419-023-06101-7)

**Original western blots**

**Figure 3G**


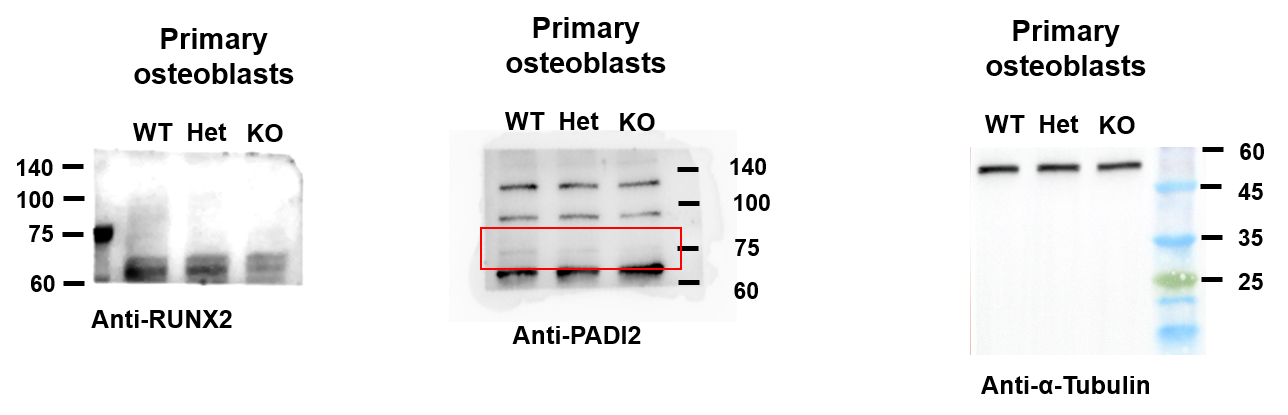


**Figure 3H**


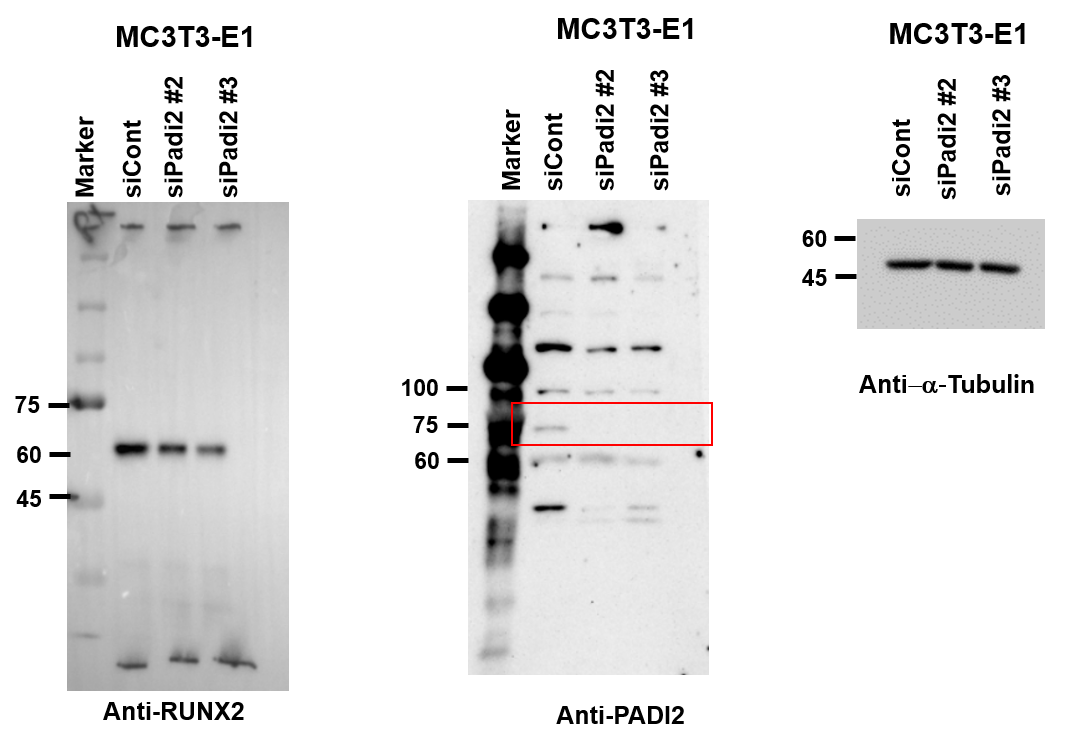


**Figure 3I**


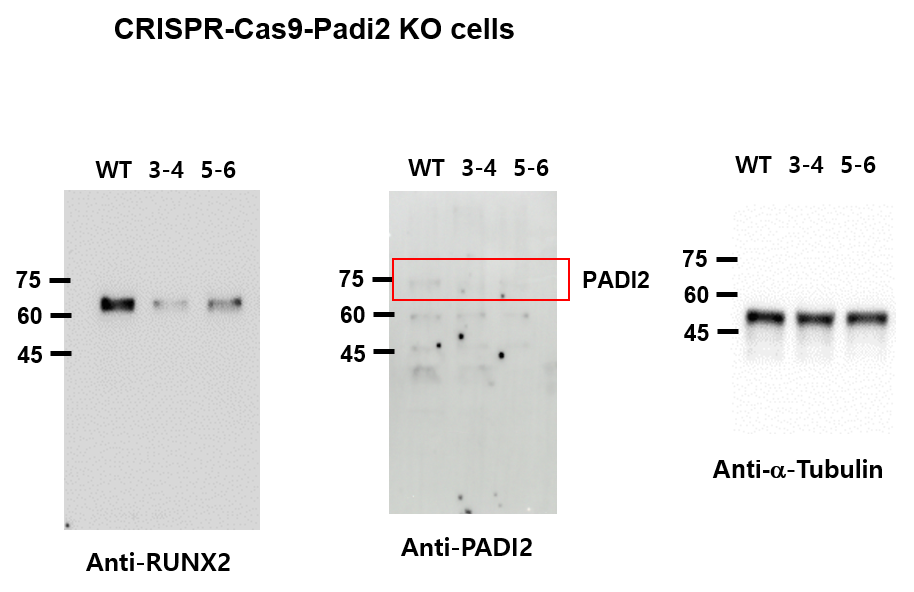


**Figure 3J**


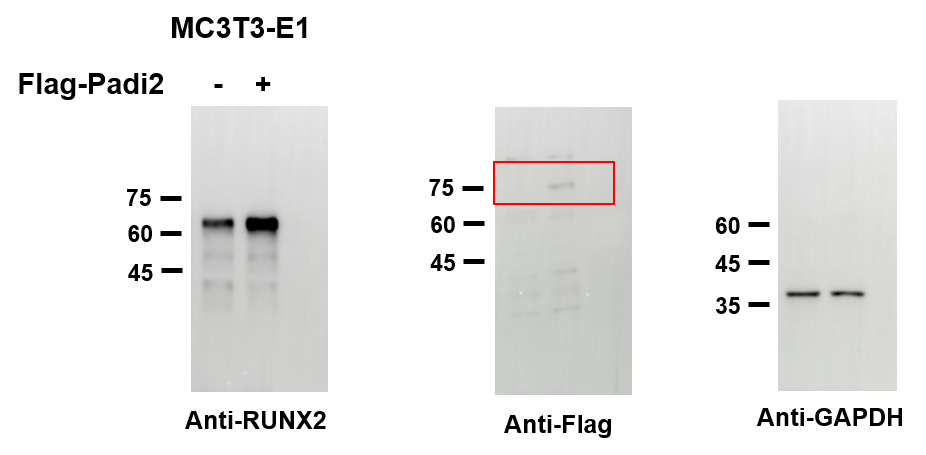


**Figure 4B**


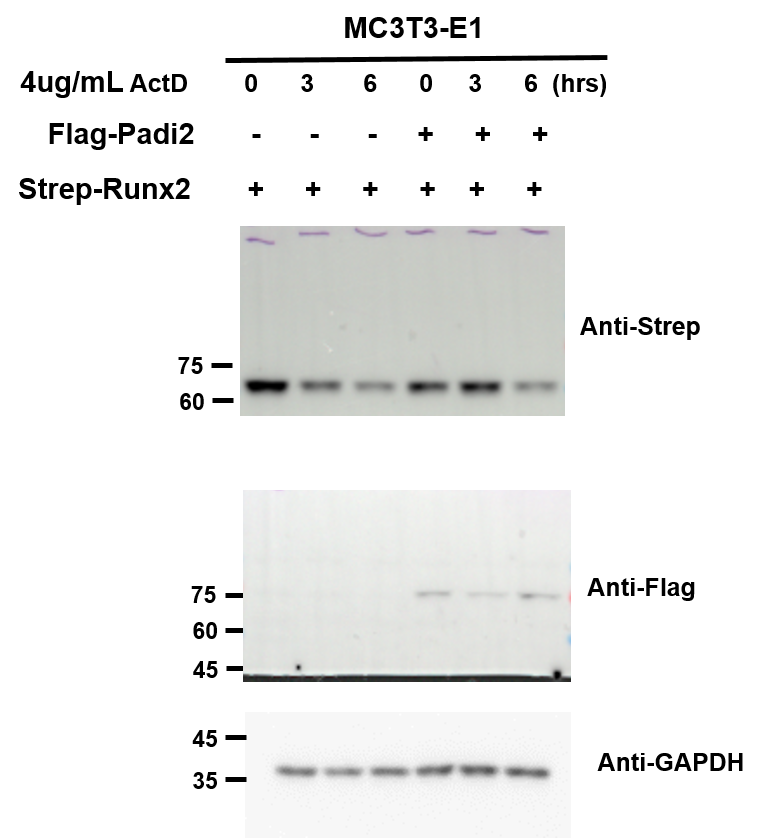


**Figure 4D**


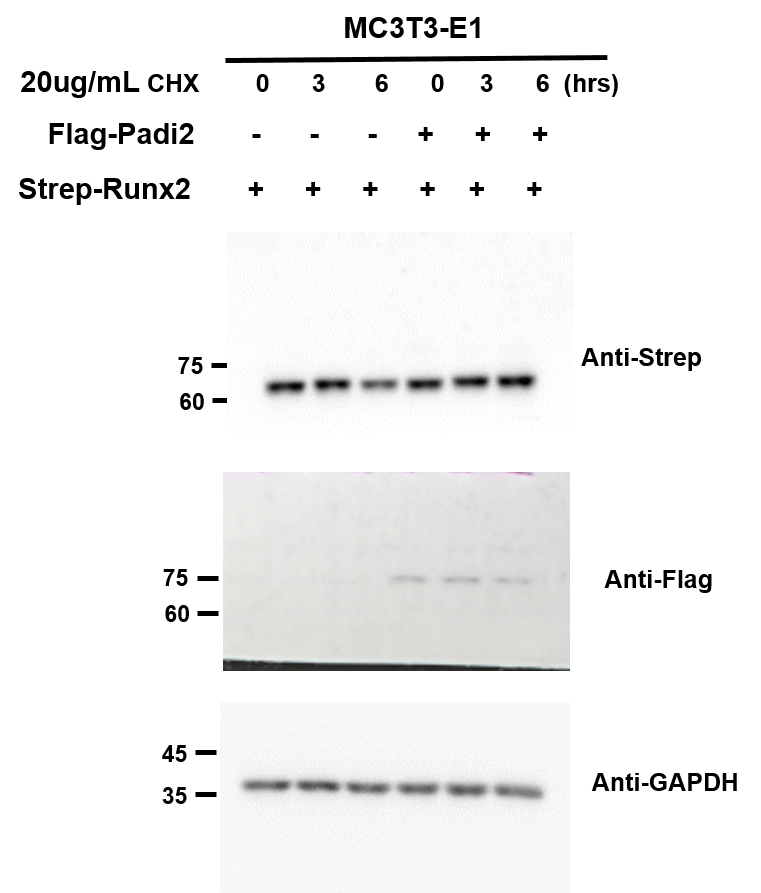


**Figure 4F**


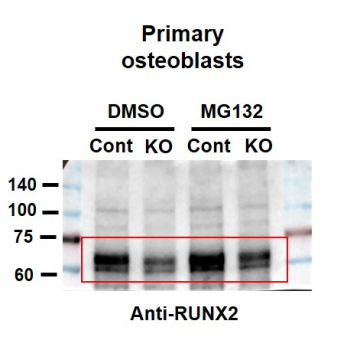

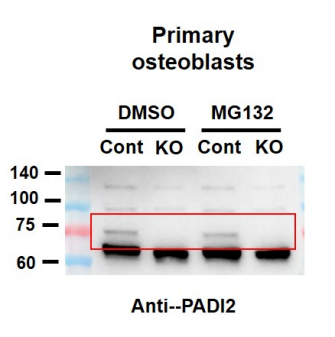


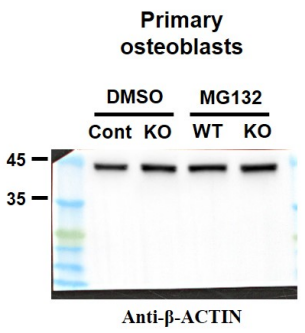


**Figure 4G**


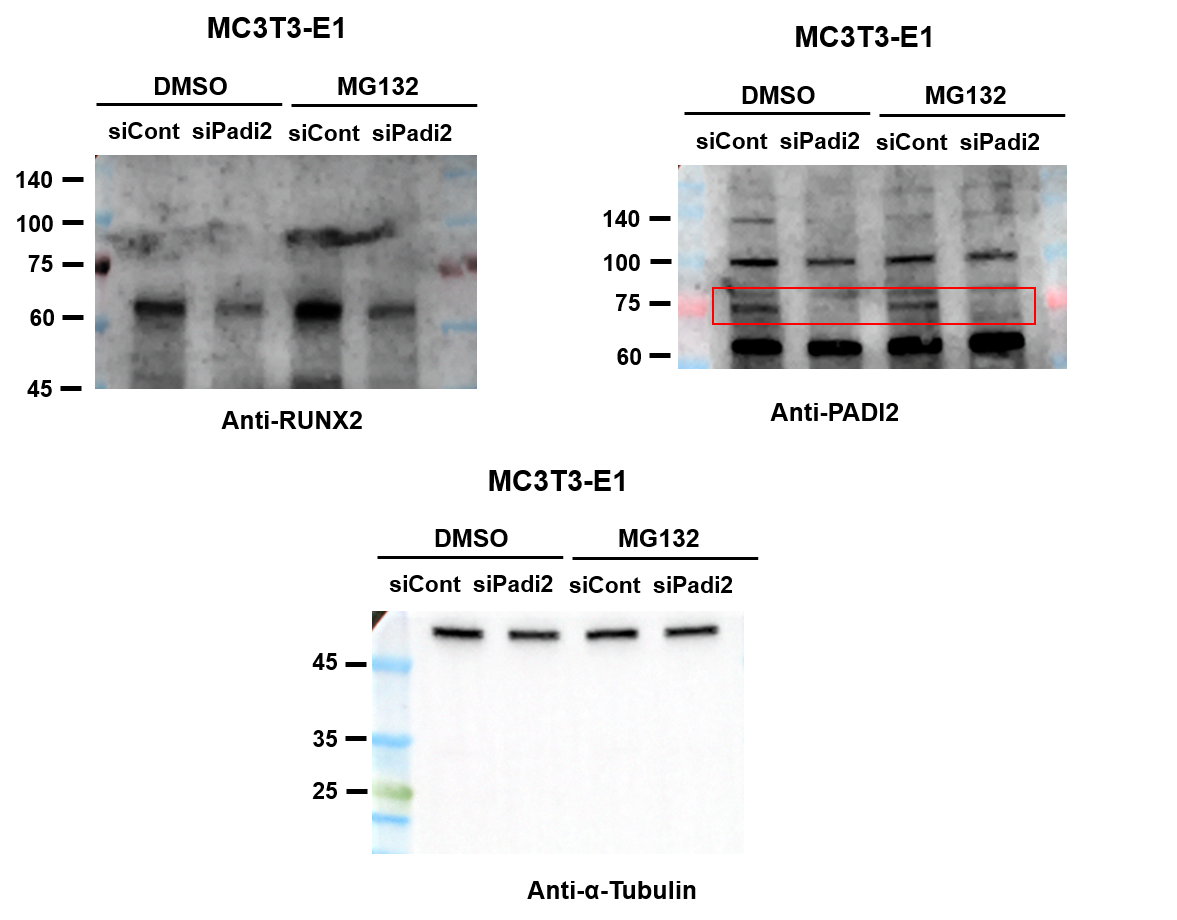


**Figure 4H**


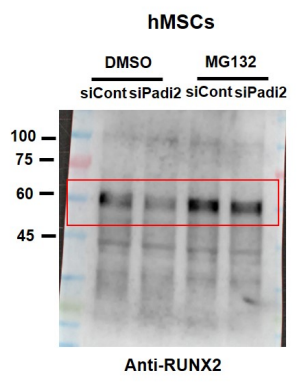

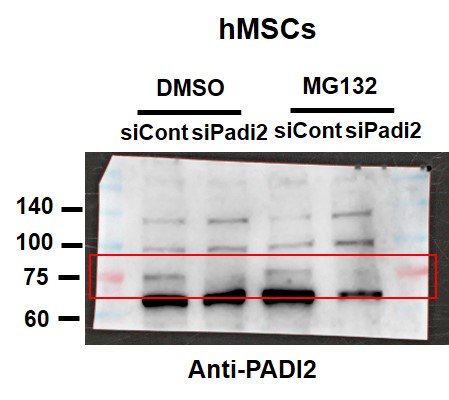


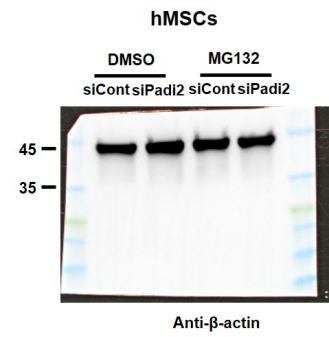


**Figure 4I**


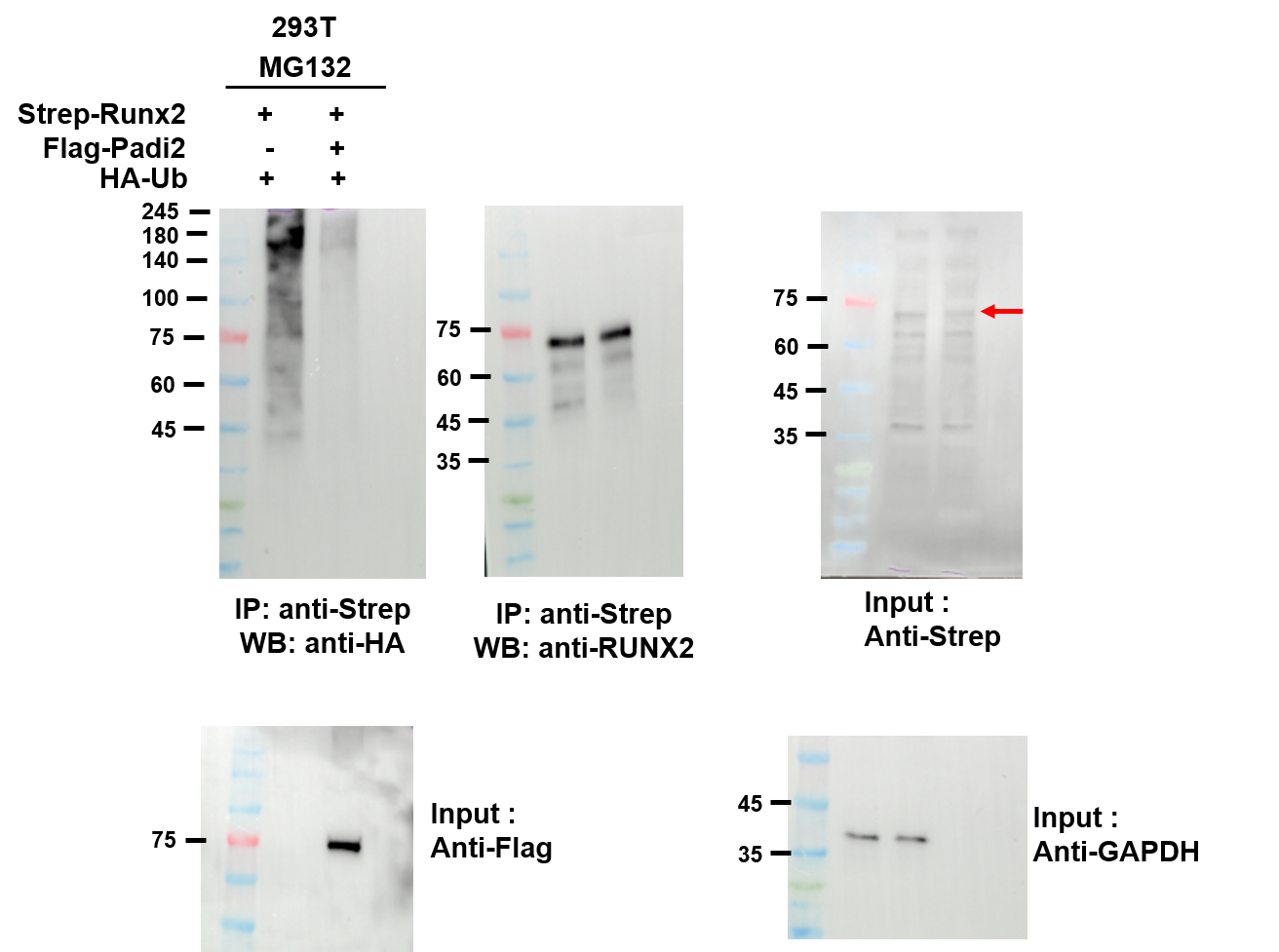


**Figure 5A**


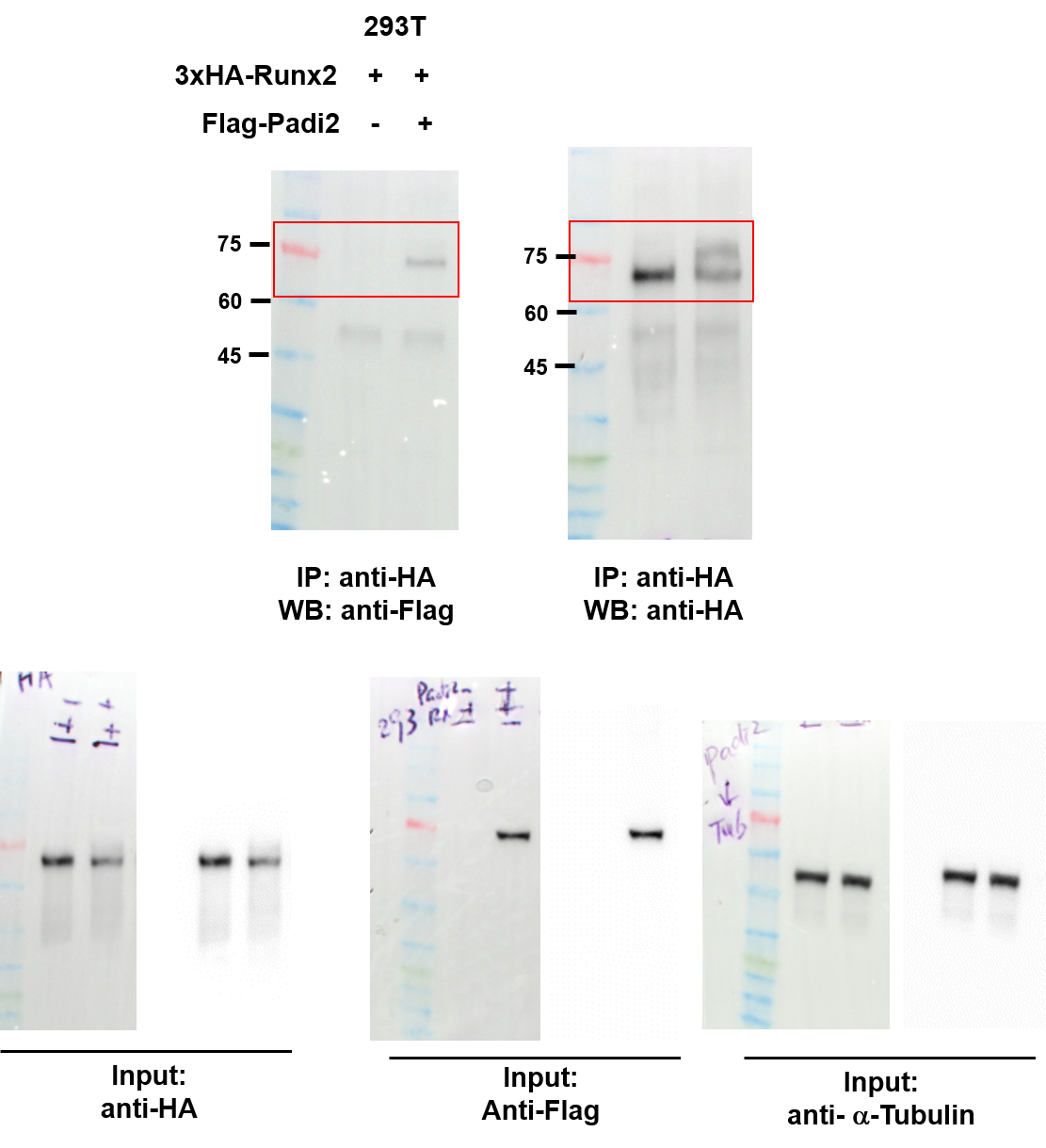


**Figure 5B**


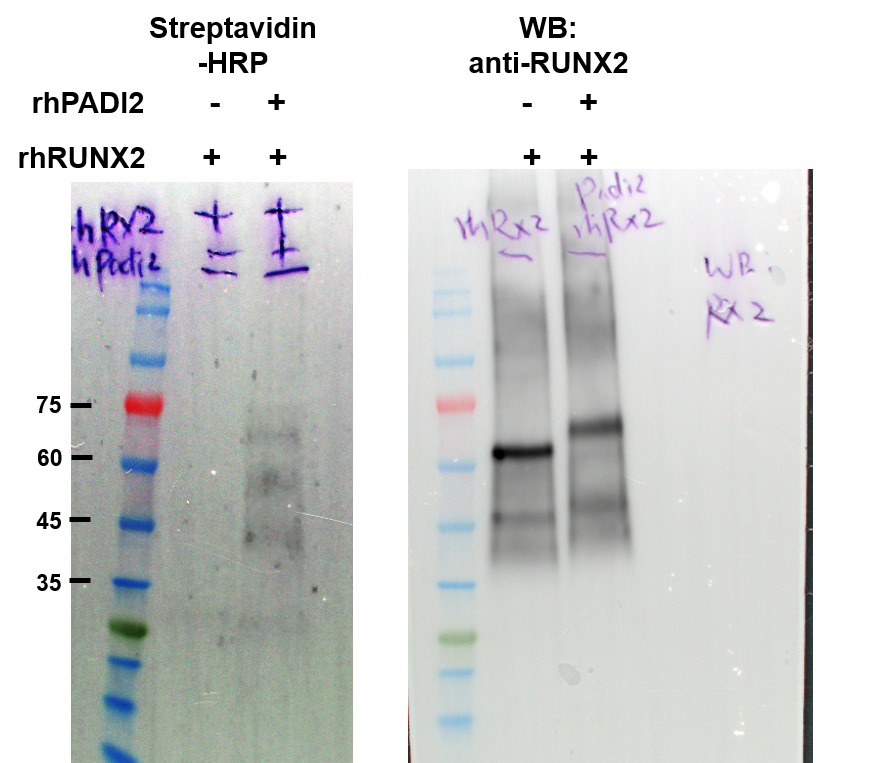


**Figure 6A**


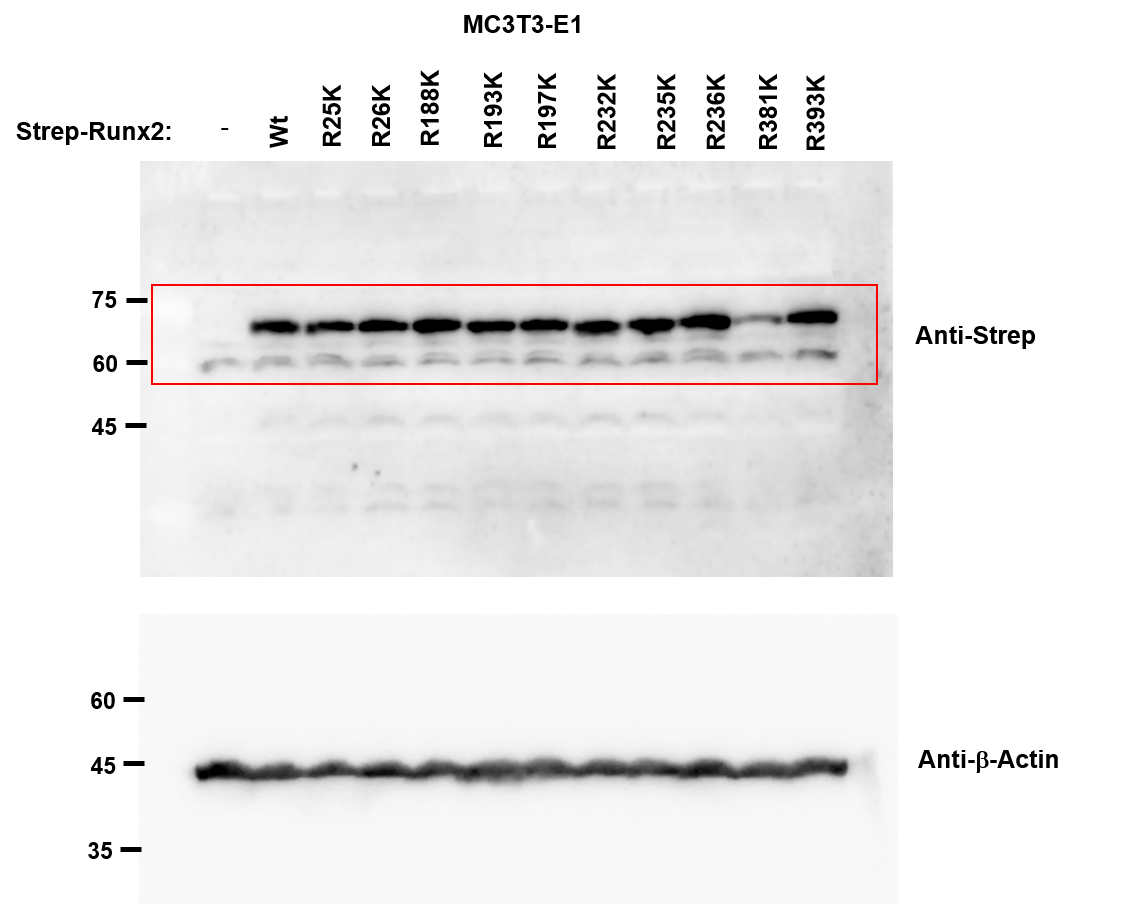


**Figure 6B**

**
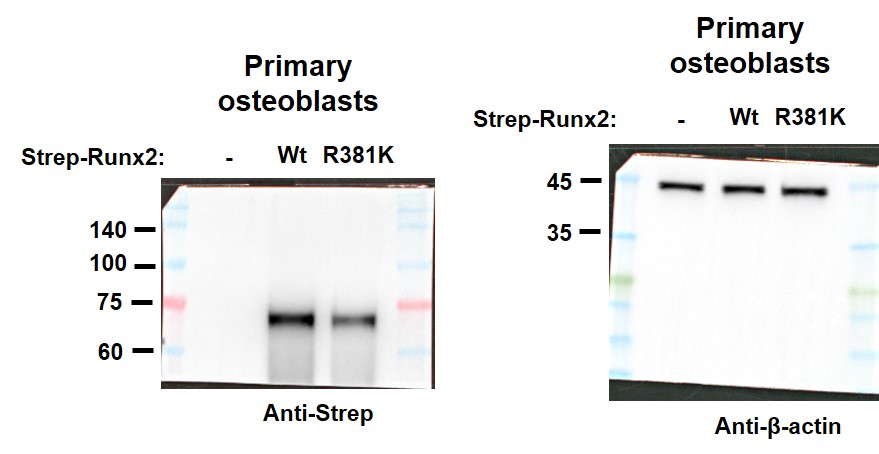
**

**Figure 6C**


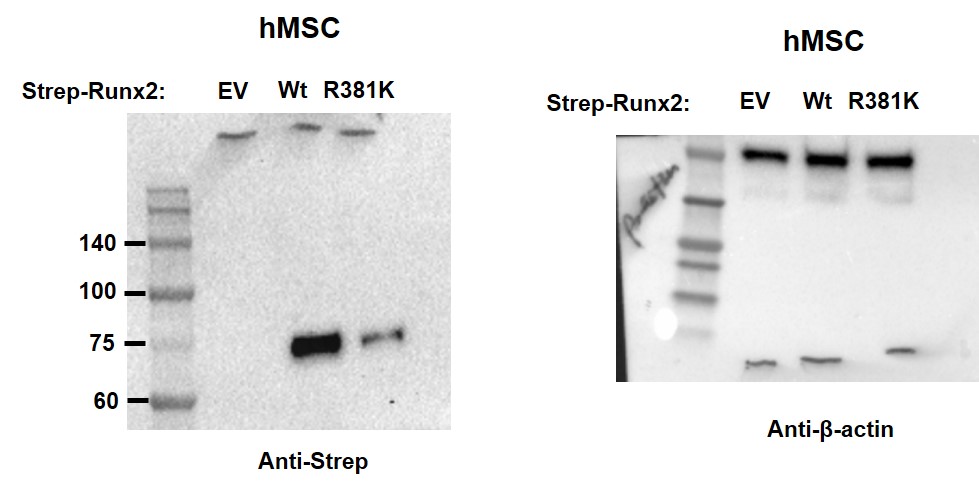


**Figure 6D**


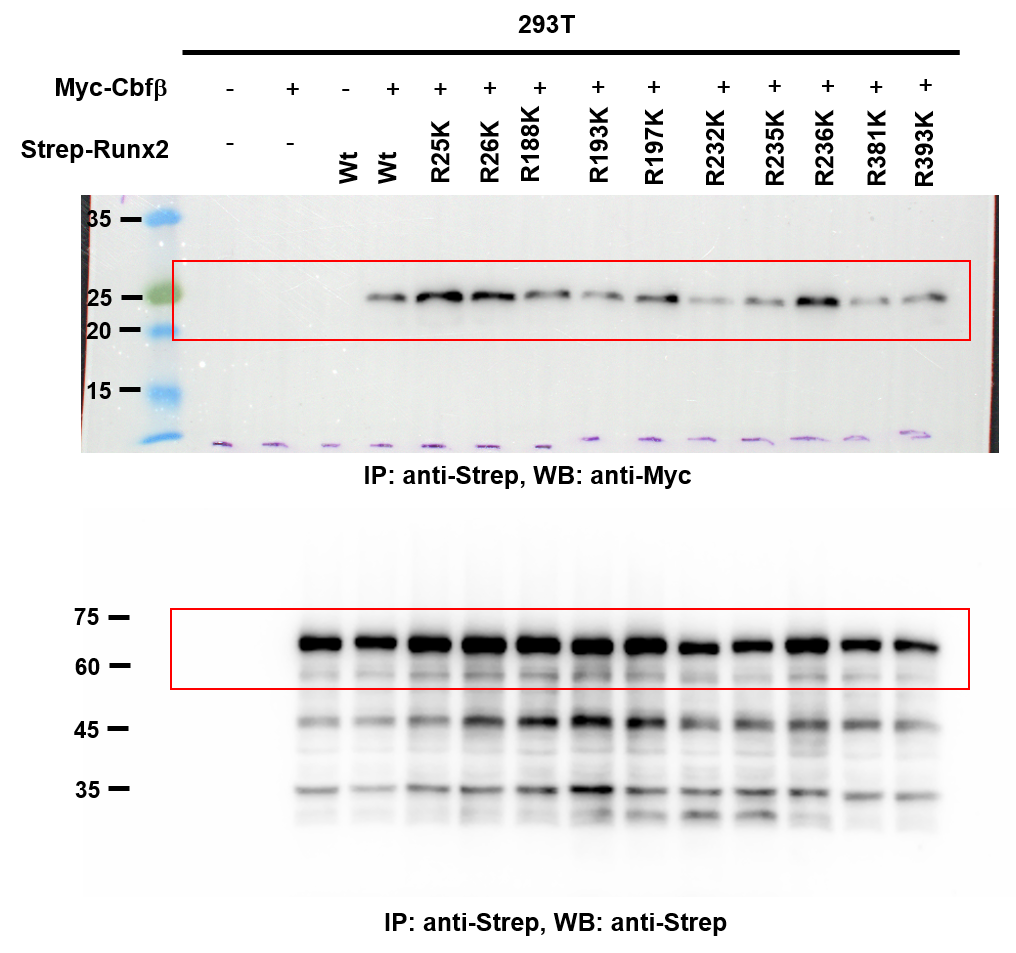


**Figure 6D continued**


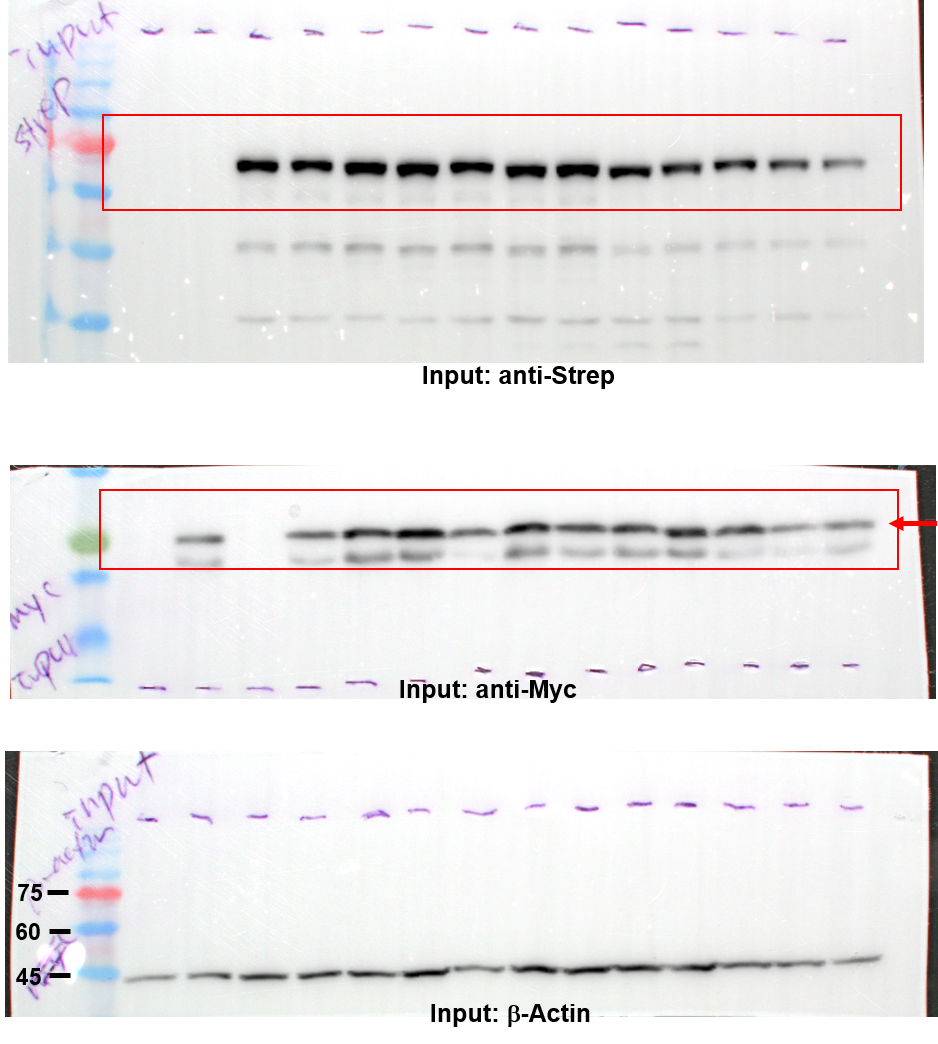


**Supplementary Figure 8**


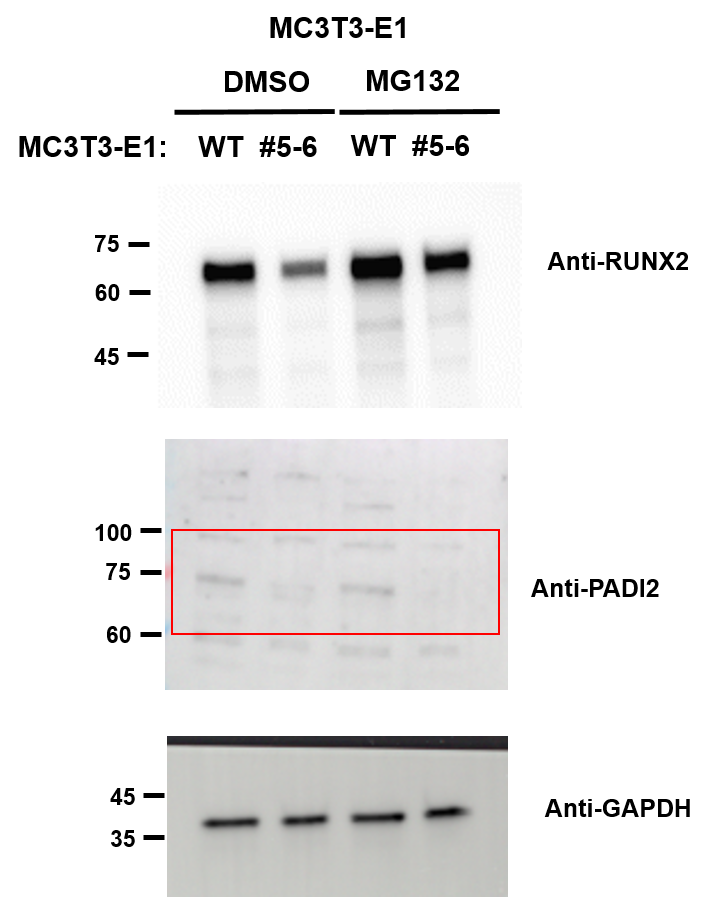

Supplement: Supplementary file 2 — Original Data File [file 41419_2023_6101_MOESM2_ESM.docx]
